# Engineered multifunctional transforming growth factor-β type II receptor ectodomain fusions for oncology applications

**DOI:** 10.3389/fonc.2025.1648779

**Published:** 2025-10-27

**Authors:** Anne E. G. Lenferink, John C. Zwaagstra, Jason Baardsnes, Laurence Delafosse, Marie Parat, Umar Iqbal, Etienne Lessard, Arsalan S. Haqqani, Anna Jezierski, Afnan Abu-Thuraia, Sébastien Tabariès, Peter M. Siegel, Traian Sulea

**Affiliations:** ^1^ Medical Devices Research Centre, National Research Council Canada, Montréal, QC, Canada; ^2^ Human Health Therapeutics Research Centre, National Research Council Canada, Montréal, QC, Canada; ^3^ Human Health Therapeutics Research Centre, National Research Council Canada, Ottawa, ON, Canada; ^4^ Department of Medicine, Goodman Cancer Institute, McGill University, Montréal, QC, Canada; ^5^ Department of Biochemistry, Microbiology and Immunology, University of Ottawa, Ottawa, ON, Canada; ^6^ Institute of Parasitology, McGill University, Sainte-Anne-de-Bellevue, QC, Canada

**Keywords:** transforming growth factor β, ligand trap, bone tumor microenvironment, epithelial-mesenchymal transition, blood-brain barrier, targeted therapy, Fc-fragment fusion

## Abstract

**Introduction:**

The transforming growth factor-β (TGF-β) superfamily consists of a large number of evolutionarily conserved and structurally related polypeptide growth factors. TGF-β elicits a wide range of context-dependent cellular responses that play important roles in the maintenance of normal physiological processes and is implicated in various pathologies, including cancer. In healthy cells and in the early stages of cancer development, TGF-β acts as a tumor suppressor by inducing cell cycle arrest and apoptosis. However, in late-stage cancer cells, TGF-β can promote tumorigenesis, including epithelial-mesenchymal transition (EMT), metastasis and chemoresistance.

**Methods:**

The dual-function and pleiotropic nature of TGF-β makes therapeutic targeting of this molecule a significant challenge. In this report, we describe the design and development of a novel class of TGF-β-targeting therapeutics in which the TGF-β type II receptor ectodomain (TβRII-ED) can be fused to an intact antibody, such as Cetuximab, or an antibody Fc fragment, without compromising the TβRII-ED or antibody function.

**Results and Discussion:**

As such, we constructed and characterized specific TβRII-ED-Fc fusions that act as efficient TGF-β ligand traps with picomolar in vitro neutralizing potencies against TGF-β1 and TGF-β3 isoforms, but not TGF-β2. We further demonstrate that TβRII-ED-Fc is a versatile ligand-trapping module that, when combined with a specific targeting moiety, can lead to powerful anticancer biotherapeutics targeted to and retained at the tumor site, by efficiently neutralizing the tumor-promoting activities of TGF-β *in vivo*.

## Introduction

The TGF-β superfamily consists of over 30 ligands that include the bone morphogenetic proteins (BMPs), growth and differentiation factors (GDFs), activins and TGF-βs (1), which control a plethora of physiological processes that take place during embryogenesis, inflammation, tissue repair, and the maintenance of adult tissue homeostasis (2). The broad range of the context-dependent cellular responses elicited by this large family and, consequently, alterations and disruptions in their signaling have been implicated in cancer and other diseases (3, 4).

There are three TGF-β isoforms (TGF-β1 (5), TGF-β2 (6) and TGF-β3 (7)), which are structurally very similar (70-80% amino-acid homology) but differ in their biological characteristics. The TGF-βs bind and activate a heterotetrameric type I and type II dual-specificity kinase receptor complex (8, 9), which triggers the phosphorylation and subsequent nuclear translocation of the Smads (10) that act as transcription factors (11, 12). Additionally, non-Smad pathways are also activated and include the Erk1/2, p38 MAP, Src tyrosine kinases, phosphatidylinositol 3- (PI3) kinases, and the Rho GTPases (13).

TGF-β signaling is a double-edged sword, as it can, depending on the stage of tumor development, inhibit as well as promote tumor growth (14). Early on, TGF-β functions as a strong anti-proliferative agent by blocking the G1 phase cell cycle progression (15), inducing apoptosis (14), regulating the production of growth factors in the surrounding stroma (16), and by inhibiting the inflammatory and immune responses (17). Nonetheless, the immuno-suppressive functions of the TGF-β family can eventually dominate the tumor microenvironment, ultimately promoting tumor growth by inhibiting cytotoxic CD8^+^ T lymphocytes (CTLs) and natural killer (NK) cells (18, 19). In addition, the induction of matrix metalloproteases and inhibitors of these further supports EMT, invasion and metastasis of tumor cells (20).

TGF-β is recognized as one of the most potent immunosuppressive factors present in the tumor microenvironment. TGF-β isoforms interfere with the differentiation, proliferation, and survival of many immune cell types, including dendritic cells, macrophages, NK cells, neutrophils, B-cells and T-cells, and thus modulates both innate and adaptive immunity (18, 21). The importance of TGF-β in the tumor microenvironment is further highlighted by evidence showing that in several tumor types, including melanoma, lung, pancreatic, colorectal, hepatic and breast, the elevated levels of TGF-β ligand are correlated with disease progression, recurrence, metastasis, and mortality. It has also been demonstrated that TGF-β is key in the inhibition of an anti-tumor response elicited by immunotherapies, such as immune checkpoint inhibitors (ICIs) (22). A therapeutic response to ICI antibodies results primarily from the re-activation of tumor-localized T-cells, and resistance to these antibodies is attributed to the presence of an immunosuppressive immune microenvironment that impairs anti-tumor T-cell mediate killing. These observations argue that in order to elicit responses in patients resistant to immune checkpoint blockade, ICI antibodies need to be combined with agents that can activate exhausted T-cells and induce their recruitment into the tumor. Overcoming this so-called “non-T-cell-inflamed” tumor microenvironment is currently the most significant hurdle in developing successful immuno-therapeutic strategies (23).

It is for these reasons that significant efforts have been invested in devising anti-tumor therapeutic approaches that involve the inhibition of TGF-β (24–26). Previously, we developed a novel protein engineering design strategy to generate single-chain, bivalent traps that, due to avidity effects, potently neutralize members of the TGF-β superfamily of ligands (WO 2008/113185; WO 2010/031168). Bivalency was achieved by covalently linking two TβRII ectodomains (TβRII-EDs) via fragments of the intrinsically disordered regions (IDR) that flank the structured, ligand-binding domain of TβRII-ED. The resulting single-chain bivalent T22d35 trap, in contrast to the monovalent non-engineered TβRII-ED (T2m) trap, potently neutralized TGF-β1 and TGF-β3, but not TGF-β2 (27, 28). The absence of TGF-β2 neutralization is considered a desirable attribute as TGF-β2 promotes hematopoiesis (29) and is crucial for normal cardiac development (30). However, despite its short serum half-life of less than 1 hour, likely due xto its 50–60 kDa size and the consequent rapid renal clearance, T22d35 was able to reverse the “non-T-cell-inflamed” tumor phenotype (28), implying that neutralization of TGF-β by T22d35 can overcome the immunosuppressive tumor microenvironment.

While the research to date indicates that single-chain TGF-β traps have promising therapeutic potential, their lack of specific tumor targeting, their short circulating half-lives and the encountered inherent manufacturability challenges, are significant challenges that prevent their development towards a potential clinical application. Hence, this manuscript describes various novel design strategies centered around recombinant fusions of the TβRII-ED (either using the T2m or T22d35 format) that address the above-mentioned challenges. For instance, we generated fusions using full-sized antibodies (e.g., with Cetuximab) or antibody Fc fragments (both C- and N-terminal fusions), made modifications to improve manufacturability and added specific targeting moieties to the Fc-fusions, such as blood-brain barrier (BBB) crossing single-domain antibodies (31) and a poly-Aspartic (D10) peptide for bone homing (32). The data presented here describe the development and functional assessment of several of these TGF-β trap fusions both *in vitro* and *in vivo*, and demonstrates the potential that these novel serum half-life-extended TGF-β ligand traps offer for the targeted delivery and retention of potent TGF-β neutralizing therapeutics at the desired site of action.

## Materials and methods

### Materials

A549 Non-Small Cell Lung Cancer cells (CCL-185; ATCC, Cedarlane Burlington ON) and HaCaT keratinocytes (CLS, Eppelheim, Germany) were grown in Dulbecco’s Modified Eagle’s Medium (DMEM) supplemented with 10% FBS. MDA-MB-468 breast cancer cells (HTB-132; ATCC, Cedarlane Burlington ON) were cultured in Leibovitz’s L15 medium supplemented with 10% FBS. All cells were maintained at 37°C in a 5% CO_2_-containing humidified environment, unless indicated otherwise.

All animal procedures were carried out in the NRC (Ottawa) and McGill University (Montréal) animal facilities accredited by the Canadian Council on Animal Care (CCAC). Studies were performed in accordance with animal use protocols approved by the NRC (AUP# 2016.06) and McGill University (AUP# 4830) Animal Care Committees and are compliant with all relevant ethical regulations regarding animal research. All mice were given food and water *ad libitum* and were housed in pathogen-free ventilated cages that were kept in a temperature-controlled room (19-21°C) with relative humidity ranging from 40-70% and under a 1h light and 1h darkness schedule.

### Trap fusion design

**Figure 1:**
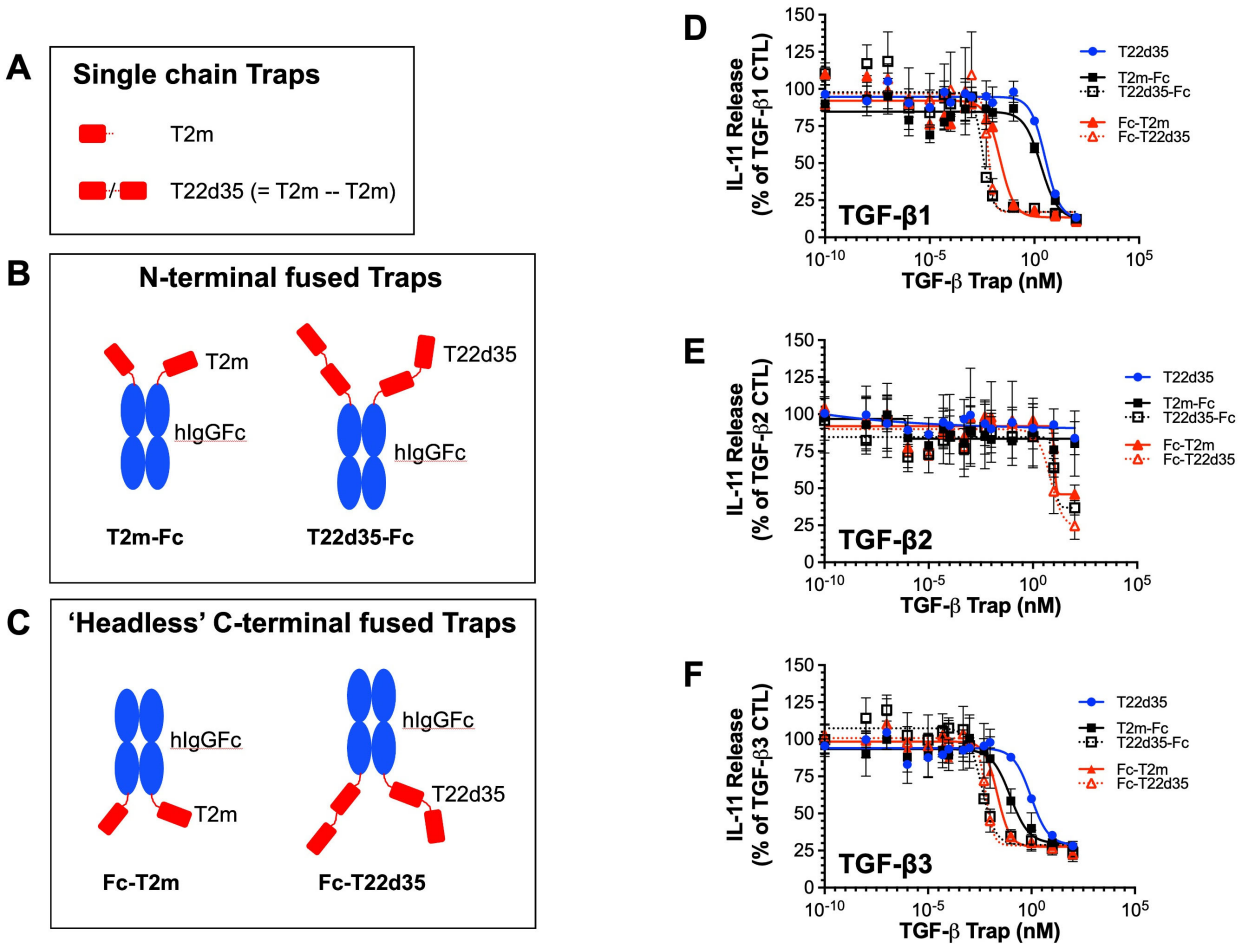
TGF-β trap design. **(A)** Schematic drawing of the TGF-β type II receptor ectodomain (TβRII-ED; abbreviated T2m) and the single-chain fusion of two T2m domains (abbreviated T22d35). Fusions of T2m and T22d35 modules to the **(B)** N-termini (T2m-Fc and T22d35-Fc) or **(C)** C-termini (Fc-T2m and Fc-T22d35) of the heavy chains of a human IgG Fc region. Red, TβRII-ED; blue, hIgG Fc fragment. Amino acid sequences of these constructs can be found in **Supplementary Tables 1 and 2**. Neutralization of **(D)** TGF-β1, **(E)** TGF-β2 and **(F)** TGF-β3 as measured in an A549 IL-11 release assay using the MSD Mesoscale platform by the N- (black; T2m-Fc, T22d35-Fc) and C-terminally Fc-fused (red; Fc-T2m, Fc-T22d35) monofunctional TGF-β Traps, compared to non-fused T22d35 (blue). Graphs show the released IL-11 in the presence of the indicated Trap fusions as the % of IL-11 released by the TGF-β1, TGF-β2, and TGF-β3 controls +/- SD. IC_50_ values (see **Table 1**) were calculated using Graphpad Prism (4-PL algorithm ((log (inhibitor) vs. response – variable slope (four parameters)).

Three-dimensional (3D) crystal structures of the TβRII-ED used for molecular design of the TGF-β traps were retrieved from the Protein Data Bank (PDB). These structures correspond to the TGF-β ligand in complex with TβRII-ED (referred to as ‘T2m’) and Fab-antigen complexes for antibodies used in the multi-functional fusions in this study. The structure of single-chain TβRII-ED dimer (referred to as ‘T22d35’) bound to the TGF-β dimer was previously predicted based on molecular dynamics (MD) simulations. Other antibody variable domain structures were modelled using the ABodyBuilder software (33). Visualizations and manipulations of molecular structures were done using the PyMol (Schroedinger, Inc.) and Sybyl (Tripos, Inc.) software. T-cell immunogenicity predictions based on peptide binding to human MHC Class-II alleles were carried out with the PROPRED software (34). Constructs encoding for monofunctional fusions in which the T2m or T22d35 (**Figure 1A**; **Supplementary Table S1**; see ref (28, 35, 36)) was fused to the N- or C-termini of the heavy chain of a human antibody IgG Fc region (**Figure 1B** (36), **Figure 1C** (35); **Supplementary Table S3**) were designed. In addition, for the N-terminal fusions the Fc hinge regions of the N-terminal fusions were engineered as shown in **Supplementary Table S3**. To further assess the trap in the context of a bifunctional fusion and to demonstrate its modularity, the trap was fused to the C-terminus of various therapeutic antibodies (Cetuximab, Herceptin, Avastin, and Synagis; **Supplementary Table S1**), while the C-terminal Fc-fused trap was N-terminally linked to a single-domain antibody (i.e., FC5V_H_H) (37) or ‘bone homing’ sequence (i.e., poly-aspartate (D10) (32, 38).

### Fusion protein expression in CHO cells

#### Monofunctional N-terminal fused T2m and T22d35 variants

Monofunctional trap Fc-fusions each contain a heavy chain Fc region and include the signal sequence (MDWTWRILFLVAAATGTHA) at their N-termini. The DNA coding regions for the constructs were prepared synthetically (Biobasic Inc. or Genescript USA Inc.) and were cloned into the *Hind*III (5’ end) and *Bam*H1 (3’ end) sites of the pTT5 mammalian expression plasmid vector (39). Fusion proteins were produced by transient transfection of Chinese Hamster Ovary (CHO) cells with the heavy chain T2m or T22d35 fused to the IgG heavy chain (T2m-HC and T22d35-HC, respectively) construct. Briefly, T2m-HC or T22d35-HC plasmid DNAs were transfected into a 2.5 L and 4.6 L culture, respectively, of CHO-3E7 cells in FreeStyle F17 medium (Invitrogen) containing 4 mM glutamine and 0.1% Kolliphor p-188 (Sigma) and maintained at 37°C. Transfection conditions were: DNA (80% plasmid construct, 15% AKT plasmid, 5% GFP plasmid) and PEI (polyethylenimine)pro (Polyplus) (ratio = 1:2.5). At 24 h post-transfection, 10% Tryptone N1 feed (TekniScience Inc.) and 0.5 mM Vaporic acid (VPA, Sigma) were added, and the temperature was shifted to 32°C to promote the production and secretion of the fusion proteins. Cultures were then maintained for 15 days post-transfection after which the cells were harvested. At the final harvest, the cell viability was 89.6%.

#### Monofunctional C-terminal Fc-fused ‘headless’, antibody-fused and bifunctional Fc-fused T2m and T22d35 trap variants

Depending on their structures, multifunctional constructs were comprised of a heavy-chain signal sequence MDWTWRILFLVAAATGTHA and a light-chain signal sequence MVLQTQVFISLLLWISGAYG (if the light chain was present in the structure). The DNA coding for constructs were prepared synthetically (Biobasic Inc. or Genescript USA Inc.). Trap constructs comprised of a “headless” Fc, antibody, and D10-Fc were cloned into the *Eco*R1 (5’ end) and *Bam*H1 (3’ end) sites and those comprising FC5-Fc were cloned into the *Hind*III (5’ end) and *Bam*H1 (3’ end) sites of the pTT5 mammalian expression plasmid vector (39). The Cet-T2m and Cet-T22d35 constructs were produced by transient co-transfection of CHO cells with the heavy chain (HC)-T2m or (HC)-T22d35 construct combined with the Cetuximab light chain (LC) construct, which then assembled as the Cetuximab-T22d35 (Cet-T22d35) or Cetuximab-T2m (Cet-T2m) fusion proteins. Briefly, CetHC-T22d35 and CetLC plasmid DNAs (ratio = 3:2) were co-transfected into a 10L Wavebag culture of CHO-3E7 cells in FreeStyle F17 medium (Invitrogen) containing 4 mM glutamine and 0.1% Kolliphor p-188 (Sigma) and maintained at 37°C. Transfection conditions were: DNA (50% HC+LC plasmids, 30% ssDNA, 15% AKT plasmid, 5% GFP plasmid): PEI (polyethylenimine)pro (Polyplus) (ratio = 1:2.5). At 24h post-transfection, 10% Tryptone N1feed (TekniScience Inc.) and 0.5 mM Vaporic acid (VPA, Sigma) were added, and the temperature was shifted to 32 °C to promote the production and secretion of the fusion proteins. Cultures were then maintained for 15 days post-transfection after which the cells were harvested. At final harvest the cell viability was 89.6%. Similar transfection and production methods were performed for the other antibody-trap examples listed in **Table 1**. For production of the ‘headless’, FC5-, and D10- Fc-fusions the composition of the transfection mixture was modified as follows: DNA (80% plasmid construct, 15% AKT plasmid, 5% GFP plasmid): PEIpro (ratio 1:2.5).

**Table 1:** Evaluation of the monofunctional Fc-fused TGF-β traps compared to the non-fused single chain T22d35 trap in the A549 IL-11 release assay (see **Figure 1**).

Monofunctional Fc-fused traps	IC_50_ (nM)		
	TGF-β1	TGF-β2	TGF-β3
T22d35	3.2530	No neutralization	0.9491
Fc-T2m	0.02293	~9.343	0.02231
Fc-T22d35	0.006297	~1.411	0.005977
T2m-Fc	2.500	~16.03	0.09430
T22d35-Fc	0.0033116	~4.763	0.003908

The IC_50_ value for TGF-β1, -β2, and -β3 was calculated using a 4-PL algorithm ((log (inhibitor) vs. response – variable slope (four parameters)) in Graphpad Prism.

### Protein purification

Similar purification methods were used for the different constructs presented here. The harvested supernatant from the CHO cells was filtered (0.2 μm) and loaded onto a Protein A MabSelect Sure column (Cytiva). The column was washed with DPBS (without Ca^2+^, without Mg^2+^, Hyclone) and protein was eluted with 0.1 M sodium citrate pH 3.6. Eluted fractions were neutralized with 1 M Tris or 1 M HEPES, and those containing the fusion proteins were pooled and subsequently desalted into DPBS using desalting columns (HiPrep 26/10, Cytiva). When required, samples were further purified by preparative size exclusion chromatography (SEC) using Superdex S200 column (Cytiva equilibrated in formulation buffer (DPBS without Ca^2+^, without Mg^2+^, Hyclone). Protein was eluted using 1 column volume formulation buffer, collected into successive fractions, and detected by UV absorbance at 280 nm. The main peak SEC fractions containing the fusion proteins were then pooled and concentrated. The integrity of the Prot-A and SEC purified fusion proteins in the pooled fractions was further analyzed by UPLC-SEC and SDS-PAGE (4-12% polyacrylamide) under reducing and non-reducing conditions (SYPRO Ruby or Coomassie brilliant blue staining). For UPLC-SEC, 2-10 μg of protein in DPBS (Hyclone, without Ca^2+^, without Mg^2+^) was injected onto a Waters BEH200 SEC column (1.7 μm, 4.6 X 150 mm) and resolved under a flow rate of 0.4 mL/min for 8.5 min at room temperature, using the Waters Acquity UPLC H-Class Bio-System. Protein peaks were detected at 280 nm (Acquity PDA detector).

### *In vitro* TGF-β neutralization

To evaluate the neutralization potency of our TβRII-ED fusions we used the A549 interleukin-11 (IL-11) release assay (40) and adapted it to the MSD Meso Scale platform, thus providing a more sensitive assay, with a better dynamic range and higher signal-to-noise ratio. Briefly, human A549 lung cancer cells were seeded in 96-well plates (5x10^3^ cells/well). The following day, 10 pM TGF-β in complete media, in the absence or presence of a serial dilution of the various TGF-β trap fusion proteins, was incubated for 30min at RT prior to adding to the cells. After 21 h of incubation (37°C, 5% CO_2_, humidified atmosphere), conditioned medium was harvested and added to MSD Streptavidin Gold plates (Meso Scale Diagnostics) that were coated with 2 μg/mL biotinylated mouse anti-human IL-11 antibody (MAB618, R&D Systems). After 18h (4°C), plates were washed with PBS containing 0.02% Tween 20 and then 2 μg/mL SULFO-tagged goat anti-human IL-11 antibody (AF-218-NA, R&D Systems Minneapolis, MN) was added, and plates were incubated for 1 h at RT. After a final wash, plates were read using the MSD QuickPlex SQ120 (Meso Scale Diagnostics). IL-11 readouts were expressed as percent IL-11 release compared to control cells treated with TGF-β alone. Experiments were carried out in triplicate and repeated at least three times; Graphpad Prism (4-PL algorithm ((log (inhibitor) vs. response – variable slope (four parameters)) was used to calculate the IC_50_.

### Competitive binding to TGF-β2 by SPR

In this assay, the trap fusions were first allowed to bind to a fixed amount of TGF-β2 in solution. Briefly, a 2-fold dilution series in PBS-0.05% Tween, starting with respectively 1000 nM T22d35 trap or 20 nM Cet-T22d35 or Cet-T2m was prepared. Each diluted sample was then pre-incubated with 1 nM TGF-β2 for 30 min at room temperature to allow binding. The mixture was then flowed over immobilized, pan-specific anti-TGF-β antibody 1D11 (2000 RU 1D11) to quantify the amount of ligand left unbound (TβRII-ED and 1D11 bind to a similar epitope on TGF-β) using a Biacore T200 instrument. The TGF-β2 binding EC_50_ values were determined by plotting the percent free TGF-β versus the protein concentration of the molecule of interest.

### Evaluation of the binding of Cetuximab-TβRII-ED fusion to the EGFR by SPR

Direct binding of Cet-T22d35 or Cetuximab to the EGF receptor extracellular domain (EGFR-ED) was quantified by SPR using a BIAcore T200 instrument, performed in the standard manner. Briefly, Cet-T22d35 or Cetuximab alone were captured on the SPR CM5 chip (BIAcore) using immobilized anti-human IgG Fc-specific antibody (2000 RU). Variable concentrations of EGFR-ED in PBS-0.05% Tween were then flowed over the capture surface at 100 μL/min at 25 °C. The resulting sensorgrams (data not shown) were analyzed using the Biacore T200 evaluation software.

### EGFR signaling

To determine the extent of EGF-induced EGFR phosphorylation in the presence of the trap fusions, A549 cells were seeded in 24-well plates (100,000 cells/well). The next day, cells were incubated in absence (CTL) or presence of Cetuximab, Cet-T2m, Cet-T22d35 or T22d35 (all at 10 nM) at 4°C for 3 h, and then treated with 50 ng/mL EGF at 37°C for 10 min. Whole-cell lysates were prepared and resolved by SDS-PAGE, proteins were transferred to nitrocellulose and probed with an anti-phospho-tyrosine antibody (Clone 4G10, Millipore 05-321) to evaluate EGFR phosphorylation levels.

### Epithelial-to-mesenchymal transition assay

A549 cells were seeded in 24-well plates (8000 cells/well) and then treated with EGF (50 ng/mL) + TGF-β1 (50 pM) at 37 °C for 3 days in the presence of Cet-T22d35, Cetuximab, or T22d35 (0, 0.05, 0.5, 5, 50, or 500 nM). Whole cell lysates were prepared and resolved by SDS-PAGE; proteins were transferred to nitrocellulose and probed with an E-cadherin antibody (BD Transduction laboratories Biosciences). E-Cadherin positive bands were quantified with a densitometer followed by analysis using ImageJ software (https://imagej.nih.gov/ij/index.html).

The ability of Cet-T22d35, Cetuximab and T22d35 to block the EGF+TGF-β induced EMT response was further examined by flow cytometry. A549 cells were seeded in 6-well plates (30,000 cells/well) and pre-treated with Cet-T22d35 (0.5 nM), Cetuximab (0.5 nM), T22d35 (1 nM) or ‘Cetuxima +T22d35’ ‘(0.5 nM+1 nM) at 37°C for 1 h, followed by addition of EGF+TGF-β1 (10 ng/mL+10 pM) and incubation at 37 °C for 3 days. Non-treated cells (without pre-treatment and EGF+TGFβ1) and cells only treated with EGF+TGFβ1 served as controls (CTL). Cells were harvested from the wells using 1 mL Dissociation Buffer (Sigma) per well, centrifuged at 2000 rpm for 2 min and re-suspended in 100 μL RPMI-5 media at 4°C. AlexaFluor488-E-cadherin (Santa Cruz, SC21791) and AlexaFluor647-N-cadherin (BD Biosciences, 563434) antibodies (1/25 v/v dilutions) were added and samples were incubated at 4°C for 1 h. Cells were then centrifuged, washed once in RPMI-5, and re-suspended in 400 μL RPMI-5 containing 15 μg/mL propidium iodide (Life Technologies) at 4 °C, after which the EMT-associated changes in cell-surface expressed E-cadherin and N-cadherin levels were quantified through measuring of the mean fluorescent intensities (MFI) by flow cytometry (BD LS RII flow cytometer, BD Biosciences).

### Cytotoxicity assay

MDA-MB-468 breast cancer cells and HaCaT keratinocytes (CLS) were seeded in 100 μL cell-specific medium at a density of 2,300 cells/well or 1,500 cells/well, respectively. The next day serial dilutions (final concentration: 0.1–100 nM) of Cetuximab, T22d35 and Cet-T22d35 were added to the wells and cells were incubated for 5 days, after which cell viability was measured using the Sulforhodamine B colorimetric assay as previously described (41). Viability was expressed as a percentage of the non-treated control, and IC_50_ values were calculated using Graphpad Prism.

### Blood-brain-barrier transport assay

SV40-immortalized Adult Rat Brain Endothelial Cells (SV-ARBEC) were used to generate an *in vitro* blood-brain barrier (BBB) model, as previously described (42, 43). Briefly, the SV-ARBECs were cultured in M199 maintenance medium (Wisent) supplemented with 10% fetal bovine serum (Thermo Fisher Scientific) and antibiotic/antimycotic (Wisent). For the BBB transport assays, the SV-ARBECs were seeded at a density of 80–000 cells onto 0.1 mg/mL rat tail collagen I (VWR)-coated permeable transwell inserts (1.12 cm² area, 1 µm pore size, Corning) in 1 mL of maintenance medium (Thermo Fisher Scientific). The basolateral companion chamber of the transwell plate contained 2 mL of maintenance medium supplemented with immortalized neonatal rat astrocytes-conditioned medium prepared in house in a 1:1 (v/v) ratio, as previously described (44). Only inserts with intact barrier formation, as assessed by a sodium fluorescein permeability value of 0.2-0.6 x 10–^3^ cm/min (as previously described (44)), were used for the BBB transcytosis studies.

### Antibody BBB transcytosis assay

For the BBB permeability assays, the SV-ARBEC inserts were transferred into companion plates containing 2 mL pre-warmed transport buffer (5 mM MgCl_2_ and 10 mM HEPES in HBSS, pH 7.4). Equimolar amounts (5.6 μM) of positive (FC5-Fc) control, negative control (A20.1), and test constructs T22d35, T2m, FC5-Fc-T22d35 and FC5-Fc-T2m were added to the top (apical) chamber of each insert and incubated with gentle rotation (20 rpm) at 37°C. Sample collections were performed at 15, 30, 45 and 60 min from the bottom (basal) wells of the companion plate for permeability analysis, as previously described (44) The protein content of each sample was then quantified by mass spectrometry (multiple reaction monitoring – isotype labeled internal standards; MRM-ILIS), as described (43). Quantified protein values were used to calculate transcytosis efficiency (percentage crossing), or P_app_ (apparent permeability coefficient) values using the following formulas, respectively:


% Transcytosis Efficiency:(output/input)×100% 

$$\text{Papp}=\frac{\text{d}Qr/\text{d}t}{\text{A }\times C_{0}}$$

The P_app_ value is commonly used to determine the specific permeability of a molecule and is a measure of transport across the brain endothelial monolayer. Qr/dt = cumulative amount in the receiver (bottom chamber) compartment versus time; A = area of the cell monolayer; C_0_ = initial concentration of the dosing solution (top chamber).

### CF-770 labelling of Fc-T2m and D10-Fc-T2m

To facilitate monitoring the behavior of the D10-trap fusions *in vivo*, we labeled the D10-Fc-T2m and the Fc-T2m control with the fluorescent CF770_NHS ester dye. Briefly, fusion proteins in PBS (pH 7.4) were diluted in 10% v/v sodium bicarbonate buffer (pH 9.3) to achieve a solution pH of 8.0. To this mixture, a 6-fold molar excess of near infrared CF770 mono-reactive NHS-ester in DMSO (Biotium Inc.) was added and allowed to react by mixing at room temperature (2h). Labeling was optimized such that each antibody had a dye/antibody ratio (DAR) of 1.5-2. After the incubation period, the protein-CF770 conjugates were purified into PBS (pH 7.4) using an Amicon 10kDa cutoff column (Millipore). DAR values were then calculated by measuring the absorbance at 280 nm (protein) and 770 nm (dye) in the linear range using a Beckman DU530 UV/Vis spectrophotometer (Beckman Coulter).

### *In vivo* imaging of CF770-labelled D10-Fc-T22d35

Male BALB/c mice (Charles River) were anesthetized on the day of the experiment using isofluorane (1.5-2%) and dorsal and ventral fur was removed by shaving followed by treatment with the hair removal cream (NAIR^®^). Mice were then injected with a single intravenous (IV) bolus of 10 mg/kg of CF770-labelled D10-Fc-T2m or Fc-T2m. Whole body bio-distribution followed over time (Prescan, 5 min, 3 h, 6 h, 24 h, 48 h, 72 h, 96 h and 120 h post-injection) using a small-animal time-domain eXplore Optix MX3 pre-clinical imager (Advanced Research Technologies (ART)). Data was recorded as temporal point-spread functions (TPSF) and fluorescence intensity map images were analyzed using the ART Optix Optiview analysis software 3.02 (ART). At the end of the imaging protocol (120 h post-injection) animals were euthanized by intracardiac perfusion using heparinized saline under deep anesthesia. Brain, heart, lungs, liver, kidneys, spleen and the right and left leg bones were dissected and imaged *ex-vivo* using the ART eXplore Optix MX3 pre-clinical imager, and images were analyzed using the ART eXplore Optix Optiview analysis software v3.02 to estimate the average fluorescence intensity in regions of interest of the dissected organs.

### Pharmacokinetic studies

Normal healthy male BALB/c animals (Charles River) were acclimatized and then intravenously (IV) injected into the lateral tail vein with a single bolus (10 mg/kg) of Cet-T22d35, D10-Fc-T2m or Fc-T2m. Blood samples were collected from the submandibular vein at selected time points (Cet-T22d35: 0 h, 0.5 h, 1 h, 2 h, 4 h, 8 h, 24 h, 48h, and 96 h; D10-Fc-T2m or Fc-T2m: 0 h, 0.25 h, 4 h, 10 h, 24 h, 48 h, 72h, 96 h, 120 h, 168 h) post-injection, centrifuged (2000*g*, 4°C, 10 min). Serum was removed, aliquoted, snap frozen on dry ice and stored at -80°C until analysis by multiple reaction-monitoring mass spectrometry (MRM-MS).

*MRM LC/MS/MS mass* sp*ectrometry:* 20 μL serum samples were thawed at 4°C, treated with mild detergents (0.1% RapiGest SF, Waters; 5.5 nM TCEP) at 95°C (10 min), cooled to room temperature (RT) and incubated (40 min in the dark) with iodoacetamide (IAA) in 50 mM ammonium bicarbonate. DTT (10 mM final concentration) was added, and the sample was incubated at RT (15 min), which was followed by trypsin digestion (Sigma, 0.8 mg/mL final concentration) at 37°C (18 h). A mixture (5μM each) of isotope-labelled trap-specific (^13^C/^15^N-(H_2_N-LPYHDFILEDAASPK-OH); further referred to as ‘LPY’ peptide) and hIgG1 Fc specific (^13^C/^15^N-(H_2_N-ALPAPIEK-OH); further referred to as ‘ALP’ peptide) specific internal standard peptides (NewEngland Peptide) in 30% acetonitrile, 0.1% formic acid) were added to a final concentration of 1 μM. Trifluoroacetic acid was added (0.5% final), followed by incubation at 37°C (30 min). Samples were then centrifuged (13,000rpm, 20 min) and the supernatant was then analyzed by MRM-ILIS on an Agilent 1260 HPLC coupled to an Agilent Triple Quadrupole LC/MS/MS (QQQ6410B) at 55°C. Final data was analyzed by a two-compartmental model using the Phoenix WinNonlin v6.3 software.

### *In vivo* efficacy of D10-Fc-T2m

For *in vivo* studies, 2.5 × 10^5^ MDA-MB-231 TR ZsGreen+ breast cancer cells were suspended in a 50:50 mixture of 1×PBS: Matrigel (Corning, 354248) and injected into the fourth mammary fat pad of 6–8-week-old female immunodeficient NSG (NOD-*Prkdc^em26Cd52^Il2rgem^26Cd22^*/NjuCrl) mice (Charles River; strain: 005557). Animals were housed in facilities managed by the McGill University Animal Resources Center and all animal experiments were conducted under an approved Animal Use Protocol (AUP#2001-4830). Twenty-four hours post mammary fat pad injection of the breast cancer cells, mice were intravenously (IV) injected via the lateral tail vein with a single bolus (10 mg/kg) of either D10-Fc-T2m, Fc-T2m or PBS as a control. Subsequent mice were injected weekly with D10-Fc-T2m and Fc-T2m at a dose of 5 mg/kg. Mammary tumors were monitored by palpation every few days and tumor volumes were calculated from weekly caliper measurements, and were harvested when tumor volumes reached between 800–1000 mm^3^. Twenty days post tumor resection, mice were imaged using X-ray microcomputed tomography (µCT). Mice were anesthetized and immobilized in the imaging 134 tube of a Skyscan 1178 µCT instrument. All images were obtained with an x-ray source operating at 45 kV (4T1) and 615 mA, with an exposure time of 480 ms. Animals were rotated through 180 degrees at a rotation step of 0.72 degrees. Cross-section images from tomography projection images were reconstructed by using the NRecon program package v.1.6.4.7 (SkyScan). Reconstruction parameters, including smoothing (1), ring artefacts reduction (1), and beam-hardening correction (30%), were fixed for all the samples. The dynamic image range was defined between 0 and 0.05 for all the samples. Bone alignment was adjusted in all specimens by using DataViewer v1.4.3.2 (SkyScan). Bone volumes were determined in 3D by using CTAn software v1.11.8.0 (SkyScan). In brief, for each bone, a volume of interest (VOI) was determined starting under the growth plate and extending 25 sections below the diaphysis. For each model, the VOI was designed by drawing user-defined polygons on the 2D sections that encompass the bone of interest. In the binary image mode, the histogram was set at minimum 100 to maximum 255 for a given dataset for each specimen. Each 3D model was visualized by using CTvox v2.3 (SkyScan). The absolute bone volume was determined for each proximal tibia and expressed in cubic millimeters, along with bone mass density expressed in grams per cubic centimeters.

### Immune system activation

#### CD4^+^ and CD8^+^ T cell proliferation

T cells were isolated from mouse spleens and seeded in 96-well plates (5x10^5^ cells/well), and co-cultured in the presence of cultured BALB/c-derived tibia bone marrow dendritic cells (DC) isolated from naïve, non-tumor bearing, untreated mice and 4T1 breast cancer cell lysate (25 μg protein per mL). DCs were obtained by flushing cells from femurs and tibias of naïve, non-tumour bearing, untreated BALB/c mice. Red blood cells were lysed using ACK lysis buffer, after which the remaining cells were cultured in complete RPMI-1640 with L-glutamine, pen/strep/β-mercaptoethanol/10% FBS supplemented with GM-CSF (10 ng/mL) and IL-4 (10 ng/mL). After 72 h, cells were further incubated for an additional 18 h in the presence of ^3^H-TdR (1 μCi), after which cells were collected onto glass fiber filters and ^3^H radioactivity was evaluated by liquid scintillography.

#### T-cell apoptosis

CD4^+^ and CD8^+^ T cells were isolated from draining lymph nodes using ThermoFisher mouse pan-T cell DynaBeads, according to the manufacturer’s instructions, stained with PE-labeled anti-CD4 and PE-CY5-labeled anti-CD8 mAbs (30 min, 4°C), washed and suspended in PBS/10%FBS/FITC-labeled Annexin V (15 min, RT). Cell populations were evaluated by flow cytometry.

#### CTL-mediated tumor cell lysis

T cells isolated from mouse lymph nodes (CyTox 96 non-radioactive cytotoxicity assay, Promega) according to the manufacturer’s instructions. Naïve target tumor cells (4T1 mouse breast or B16F10 mouse melanoma cells) were plated and incubated for 4 h with CD8^+^ effector T cells isolated (as describe above) from the lymph nodes of mice treated with saline, T22d35, or T22d35-Fc. The LDH release by the tumor cells in response to the T cells was evaluated over a period of 30 min using various effector (E) and target (T) cell ratios (E:T=10:1, 25:1, and 50:1) by measuring the LDH-mediated reduction of INT dye to blue formazan according to the manufacturer’s instructions (LDH cytotoxic assay kit, Abcam).

## Results

### Design and selection of lead monofunctional N- and C-terminal Fc-fused and bifunctional traps

Our original single-chain TGF-β trap was found to be a potent TGF-β1 and -β3 neutralizer albeit with a very short serum half-life of only 1 h (28). To solve this issue, we designed a set of monofunctional and bifunctional trap fusions in which the T2m and T22d35 is fused to either the C- or N-terminus of various hIgG Fc fragments, or the C-terminus of full-size antibodies or other targeting entities.

#### Monofunctional C-terminal Fc trap fusions

We initially linked the T2m module to the C-terminus of a hIgG1, -2, -3, and -4 Fc-fragments via the TβRII natural linker sequence. It should be noted that the hinge regions of the four hIgGs differ in terms of their cysteine content; the hIgG1 and hIgG4 hinges contain 2 cysteines while the hIgG2 and hIgG3 hinges contain, 3 and 11 cysteines, respectively. The bare N-terminal Fc hinge regions of the so-called ‘headless’ trap fusions could, if left unaltered, negatively affect protein expression and cause aggregation during the manufacturing process. We therefore engineered these hinge regions by 1) removing (ΔC) the cysteines altogether or 2) replacing cysteines by serines (S). For the hIgG3 fusions, due to its long hinge region, we made these modifications using a truncated Fc fragment which contains only the last 3 cysteine residues in its hinge region. Using this approach, the hIgG3 fusions thus contain a hinge region that closely matches the hinge region of the other human IgGs (**Supplementary Table S2**). All fusions could be produced and purified, except for those containing the hIgG4 Fc, with yields that can be ranked as follows: hIgG1 > hIgG2 > hIgG3 (**Supplementary Table S7**). This allowed us to select hIgG1Fc(SCC)ΔK-T2m (indicated in bold in **Supplementary Table S2**). However, to avoid any potential immunogenicity issues caused by the serine residues, we further engineered the N-terminal sequence by deleting the ‘EPKSS’ sequence segment from its hinge, thus generating our hIgG1Fc(CC)ΔK-T2m lead. Building on the knowledge obtained through the N-terminal designs, we then devised three C-terminal T22d35 Fc-fusions: hIgG1Fc(C)ΔK-T22d35, hIgG1Fc(CC)ΔK-T22d35, and hIg2Fc(CC)ΔK-T22d35 (**Supplementary Table S3**). Of these three, the hIgG2Fc(CC)ΔK-T22d35 production showed the highest monomeric content (96.75%), the lowest level of aggregation (3.3%) and contained no fragments, compared to the other two fusions (**Supplementary Figure S2**). We thus selected the hIgG1Fc(CC)ΔK-T2m and hIg2Fc(CC)ΔK-T22d35 as our C-terminally Fc-fused leads for further development, which are subsequently referred to as Fc-T2m (bold in **Supplementary Table S5**) and Fc-T22d35 (bold in **Supplementary Table S3**), respectively.

#### Monofunctional N-terminal Fc trap fusions

In addition to the C-terminal Fc-fusions we also devised one T2m and five T22d35 N-terminally hIgG1 and -2 Fc-fusions while applying a variety of linker designs (**Supplementary Table S4**). All fusions were produced and purified, and showed a monomeric content of >98%, with a low % of aggregates, and no fragment content (**Supplementary Figure S2**). Of these variants we selected the T2m-hIgG2Fc(CCCC)ΔK and the T22d35-hIgG2Fc(CC)ΔK as our N-terminally Fc-fused leads for further assessment; these variants are, further referred to as T2m-Fc and T22d35-Fc, respectively (bold in **Supplementary Table S4**). **Supplementary Figure S1** shows the purification profiles of the selected C-terminal Fc-T2m (A) and Fc-T22d35 (B) and N-terminal T2m-Fc (C) and T22d35-Fc (D) lead fusions. Size exclusion Chromatography (SEC) elution profiles after Protein-A affinity purification revealed that these fusions are relatively pure and devoid of aggregates, and were shown to be >95% monomeric by UPLC-SEC, with the exception of the T22d35-Fc, which is ~87% monomeric. Further SDS-PAGE assessment, confirmed the expected molecular weight of these fusions: ~60 kDa and ~90 kDa for Fc-T2m and T2m-Fc, and ~90 kDa and ~150 kDa for Fc-T22d35 and T22d35-Fc, under respectively reducing and non-reducing conditions, respectively.

#### Bifunctional trap fusions

The purpose of this manuscript is also to demonstrate the modularity and potential therapeutic functionality of TGF-β trap in the context of a bifunctional moiety by fusing the trap to N-terminus of several antibodies or other functional entities such as a blood-brain barrier (BBB) crossing (FC5V_H_H) (37) or a bone targeting moiety (D10) (32, 38). For the generation of bifunctional antibody-trap fusions (**Supplementary Table S5**), we used the well-characterized monoclonal antibodies Cetuximab, Herceptin, Avastin and Synagis, which all showed very similar production and purification profiles. **Supplementary Figure S1** shows the purification profile of Cet-T2m (E) and Cet-T22d35 (F) as representative examples, showing a monomeric purity of these fusions by UPLC-SEC of >99% after Protein-A and SEC purification, and their expected molecular weight by SDS-PAGE [Cet-T2m: ~242 kDa (non-reducing), and ~78 kDa (HC) and ~27 kDa (LC) under reducing conditions; Cet-T22d35: ~250 kDa (non-reducing), and ~110 kDa (HC) and ~30 kDa (LC) under reducing conditions].

To generate other bifunctional non-antibody trap fusions, we C-terminally linked the T2m and T22d35 to an already existing N-terminally mIgG2a Fc-fusion of the single domain FC5V_H_H (37), and we also fused a functional ‘homing’ peptide, the D10 poly-aspartate sequence (32, 38), to the N-terminus of the earlier described Fc-T2m (**Supplementary Tables S5 and S6**). It should be noted that the hinge region of the mIgG2a Fc in the FC5V_H_H fusions was not re-engineered. Four D10 fusion were designed using various sequences linking the D10 to the N-terminus of the Fc-T2m (**Supplementary Table S6**). These fusions were expressed at a small scale with very similar yields and monomeric purities >98% (data not shown), indicating that neither the D10, nor the linker sequences used were detrimental for the production and purification of this fusion. We selected the D10-hIgG1Fc(CC)ΔK-T2m fusion for further functional assessment.

The selected FC5V_H_H-Fc-T2m, FC5V_H_H-Fc-T22d35 (**Supplementary Table S5**) and D10-FchIgG1(CC)ΔK-T2m (**Supplementary Table S6**) fusions are, from this point, referred to as FC5-Fc-T2m, FC5-Fc-T22d35 and D10-Fc-T2m, respectively. **Supplementary Figure S1** shows the purity of the FC5-Fc-T2m (G), FC5-Fc-T22d35 (H) and D10-Fc-T2m (I) to be >94%, 83%, and >99% monomeric by UPLC-SEC after Protein-A and SEC purification, and their expected molecular weight by SDS-PAGE [FC5-Fc-T2m: ~100 kDa (non-reducing), and ~80 kDa under reducing conditions; FC5-Fc-T22d35: ~155 kDa (non-reducing), and ~80 kDa under reducing conditions; D10-Fc-T2m: ~150 kDa (non-reducing), and ~80 kDa under reducing conditions]. Duplicate bands are likely the result of differences in glycosylation.

An overview of the production and purification for all antibody-, FC5- and D10-trap fusions that were generated for this study is shown in **Supplementary Table S9**. Our data clearly demonstrates that, using the described design strategies, our T2m and T22d35 traps, either as a N- or C-terminal fusion in the context of an Fc fragment alone, combined with an additional targeting moiety, or as an antibody can be produced at relatively high titers and can be purified by Protein-A followed by SEC. This demonstrates good manufacturability of both the mono- and bifunctional T2m and T22d35 N- and C-terminally Fc-fused proteins. It should be noted that the C-terminal lysine residue of the Fc fragment was deleted from all trap fusions to avoid cleavage of C-terminal linked sequences.

### Functional evaluation of the T2m and T22d35 Fc-fusions

To assess functionality of the Fc-fused TGF-β traps, we assessed their neutralizing effects and compared them to that of the T22d35 single-chain divalent trap in the TGF-β-induced A549 IL-11 release assay.

#### Monofunctional T2m and T22d35 Fc-fusions

For all TGF-β isoforms (**Figure 1D**, TGF-β1; **Figure 1E**, TGF-β2; **Figure 1F**, TGF-β3), the potency of both N- and C-terminally Fc-fused T22d35 and the N-terminally fused Fc-T2m is superior to that of the C-terminally fused Fc-T2m, which in turn behaves similarly to the non-Fc-fused T22d35 single-chain trap. Monofunctional Fc-fused traps can therefore be ranked as follows: Fc-T22d35 ≈ T22d35-Fc > Fc-T2m > T2m-Fc ≈ T22d35. The calculated IC_50_ values against TGF-β1 (**Table 1**) for Fc-T22d35 and T22d35-Fc (0.006297 nM and 0.0033116 nM) demonstrate their potencies to be at least ~520- and ~920-fold better than the IC_50_ values calculated for T22d35 (3.253 nM). When comparing their TGFβ1 IC_50_ to those calculated for Fc-T2m (0.02293 nM) and T2m-Fc (2.500 nM), the Fc-T22d35 is ~3.6-fold better than the Fc-T2m while the T22d35-Fc is ~760-fold more potent than the T2m-Fc. This indicates that the orientation of the trap, with respect to the hIgG Fc fragment, is very important for its TGF-β1 neutralization. In addition, our data also shows that Fc-T22d35 and T22d35-Fc neutralize TGF-β2 more effectively compared to either the Fc-T2m or T2m-Fc constructs; however, this is still to a much lesser extent than TGF-β1 and -β3. It should be noted that, although the neutralization potency of the Fc-T22d35 and T22d35-Fc trap are very similar for TGF-β1 and -β3, the T2m-Fc variant displayed a ~27-fold higher neutralization potency for TGF-β3 compared to TGF-β1 (0.0943 nM versus 2.500 nM, respectively), suggesting that the T2m conformation as an N-terminal Fc-fusion is better suited to neutralize TGFβ-3 compared to TGF-β2. Moreover, the neutralization potency of the C-terminal T2m and T22d35 fusions, and to a lesser extend the N-terminal fusions, significantly increased compared to the non-fused T2m and single-chain non-fused T22d35 protein, which are non-neutralizing with respect to TGF-β2 (28). Fusing more than two T2m domains to an Fc fragment (i.e. more than the two T2m domains that are present in the T22d35 construct) did not further increase the TGF-β1 neutralization potency of these fusions when compared to Fc-fused T22d35 (data not shown).

#### Antibody-T2m and -T22d35 fusions

**Figure 2:**
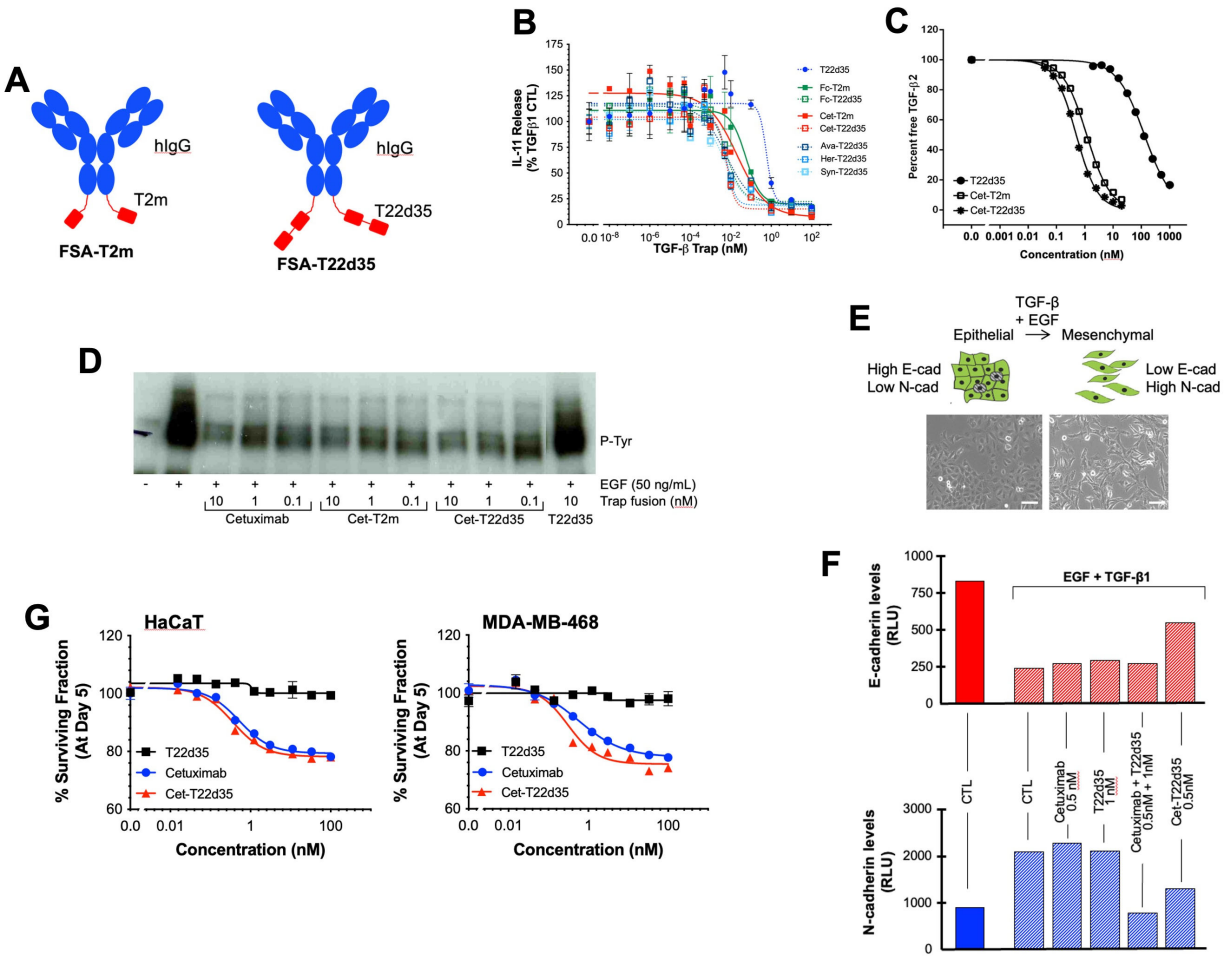
Design and *in vitro* functional evaluation of full-size antibody fused TGF-β traps. **(A)** Schematic drawing of the TβRII-ED-based bifunctional full-size antibody (FSA) trap design: FSA-T22d35 Trap (right) and FSA-T2m fusions (left) (Red, TβRII-ED; blue, FSA). **(B)** Neutralization of TGF-β1 by the FSA-T2m and FSA-T22d35 (FSA = Cetuximab (Cet), Herceptin (HER), Avastin (AVA) or Synagis (SYN)), compared to the monofunctional T22d35 single chain, Fc-T2m and Fc-T22d35 traps assessed in the A549 IL-11 release assay using MSD Mesoscale platform. Graphs show the released IL-11 in the presence of the indicated Trap fusions as the % of IL-11 released by the TGF-β1 control +/- SD. IC_50_ values (see **Table 2**) were calculated using Graphpad Prism (4-PL algorithm ((log (inhibitor) vs. response – variable slope (four parameters)). **(C)** Graph showing the competitive SPR analysis of the binding of TGF-β2 (1 nM fixed concentration) to a 0.02–20 nM serial dilution of Cet-T2m (open square) and Cet-T22d35 (star), and 3.9–1000 nM serial dilution of non-fused T22d35 (closed circle). IC_50_ values (see text) were calculated using Graphpad Prism (4-PL algorithm ((log (inhibitor) vs. response – variable slope (four parameters)). **(D)** Western blot analysis showing EGFR phosphorylation (anti-P-Tyr) in non-treated (-), and EGF-treated (+; 50 ng/mL) A549 cells in the absence (CTL) and presence of 0.1–10 nM Cetuximab, Cet-T2m, Cet-T22d35, or 10 nM T22d35. **(E)** Cartoon (top) and microscopic pictures (bottom) depicting cells undergoing an EMT upon exposure to EGF+TGF-β1, changing from a ‘cobble-stone’ epithelial to the elongated mesenchymal morphology (scale bar = 100 µm). **(F)** Flow cytometric assessment of the E- and N-cadherin cell surface expression levels in A549 cells undergoing an EGF+TGF-β1 induced EMT in the presence or absence of the indicated Trap fusions. **(G)** Survival curves of MDA-MB-468 (top) and HaCaT (left) cells after 5-day exposure to serial dilutions of T22d35 (black squares), Cetuximab (blue circles) and Cet-T22d35 (red triangles). Cell viability was assessed by Sulforhodamine B and expressed as a % of non-treated cells +/- SD. IC_50_ values (see **Table 4**) were calculated using Graphpad Prism (4-PL algorithm ((log (inhibitor) vs. response – variable slope (four parameters)).

To demonstrate the modularity of the T2m and T22d35 traps we generated a series of bifunctional antibody fusions in which the C-terminus of the heavy chain was linked to the trap (**Figure 2A**; Cet, Cetuximab; Her, Herceptin; Ava, Avastin; Syn, Synagis), and evaluated both the antibody and trap function in the context of the fusion.

#### TGF-β1 neutralization

To confirm the relative TGF-β neutralization of antibody fusions, we used the earlier described A549 IL-11 release assay. In these experiments (**Figure 2B**), the TGF-β1 neutralization potencies of Cet-T2m and Cet-T22d35, Her-T22d35 and Syn-T22d35 were compared to T22d35, Fc-T2m and Fc-T22d35. As anticipated, the neutralization potencies of all antibody -T22d35 fusions were practically identical to that of the C-terminal Fc-T22d35 fusion (**Table 2**), exhibiting IC_50_ values in the range of 0.0005 nM, whereas the IC_50_ value for the T2m fusions (e.g., Cet-T2m) and the non-Fc-fused T22d35 were calculated as ~0.05 nM and ~0.5 nM, respectively. As such antibody -T22d35 and Fc-T22d35 fusions displayed a ~100-fold increase in TGF-β1 neutralizing potency, but also demonstrated that constructs with two C-terminally fused TGF-βRII-EDs (i.e., T22d35) are ~10-fold more potent than similar constructs containing a single TGF-βRII-ED (i.e., T2m).

**Table 2:** Evaluation of the bifunctional FSA-fused TGF-β traps in the A549 IL-11 release assay.

Bifunctional FSA fused traps	IC_50_ (nM)		
	TGF-β1	TGF-β2	TGF-β3
Cet-T2m	0.057200	~9.0742	0.03320
Cet-T22d35	0.006124	~3.3321	0.00428
Her-T22d35	0.006456	~4.3457	0.00249
Ava-T22d35	0.005671	~2.9931	0.00276
Syn-T22d35	0.004398	~3.1286	0.00281

The IC_50_ value for TGF-β1, -β2, and -β3 was calculated using a 4-PL algorithm ((log (inhibitor) vs. response – variable slope (four parameters)) in Graphpad Prism.

#### Antibody-antigen binding

We then evaluated the binding of the Cet-T22d35, Her-T22d35 and Syn-T22d35 fusions to their intended target antigen by SPR, by comparing these to the non-fused antibody s. Our data shows that the K_D_ values of al antibody fusions are very similar to the ones calculated for the respective non-fused parental antibodies (**Table 3**), clearly demonstrating that fusion of one (Cet-T2m) or two (Cet-T22d35, Her-T22d35, or Syn-T22d35) TGF-βRII-ED(s) to the C-terminus of an antibody Fc region does not significantly alter antigen-binding affinities and K_D_ values of the antibody. These data suggest that the ectodomain(s) can be readily fused to any antibody without compromising the ability of the antibody to bind its target antigen.

**Table 3:** Evaluation kinetic parameters by SPR of the antigen-binding affinity for the various FSA-T22d35 fusions (with the exception of Avastin) compared to their respective parental antibody.

		Kinetic Parameters Antigen Binding		
	Antigen	K_a_ (1/Ms)	K_d_ (1/s)	K_D_ (M)
Cet-T2m	EGFR	1.34 X10^6^	8.51 X10^-4^	7.39 X10^-10^
Cet-T22d35	EGFR	1.22 X10^6^	8.65 X10^-4^	7.08 X10^-10^
Cetuximab	EGFR	1.03 X10^6^	8.45 X10^-4^	8.47 X10^-10^
Her-T22d35	Her2	8.30 X10^4^	5.30 X10^-5^	6.37 X10^-10^
Herceptin	Her2	6.88 X10^4^	5.03 X10^-5^	7.33 X10^-10^
Syn-T22d35	RSV-F	3.55 X10^4^	1.42 X10^-3^	4.10 X10^-9^
Synagis	RSV-F	2.57 X10^4^	1.68 X10^-3^	6.60 X10^-9^

#### In solution TGF-β2 binding

To gain insight into whether the binding of TGF-β2 was affected when the TβRII-ED was fused to an antibody, we analyzed TGF-β2 binding by Cet-T2m, Cet-T22d35, and T22d35 in a competitive SPR binding experiment (**Figure 2C**). Our data clearly shows that the binding of TGF-β2 by TβRII-ED dramatically increases when it is fused to the C-terminus of an antibody such as Cetuximab, with a ~200-fold and ~100-fold increase in the TGF-β2 EC_50_ values for Cet-T22d35 (EC_50_ = 0.50 nM) and Cet-T2m (EC_50_ = 1.17 nM), respectively, when compared to non-fused T22d35 (EC_50_ > 100 nM). This indicates that the antibody fusion of either T2m or T22d35 improves the affinity for TGF-β2, and agrees with the increased degree of TGF-β2 neutralization we observed for Fc-T2m and Fc-T22d35 fusions in the A549 IL-11 neutralization assay (**Table 1**).

To further demonstrate that the antibody function *per se* is not affected by the presence of C-terminally fused TGF-βRII ectodomain(s), we further evaluated other attributes of the Cetuximab fusions (using Cet-T2m and Cet-T22d35 as examples).

#### Evaluation of the EGFR inhibition

To assess the ability of Cetuximab to maintain its therapeutic function when fused to either T2m or T22d35, we evaluated the Epidermal Growth Factor (EGF)-induced phosphorylation of the Epidermal Growth Factor Receptor (EGFR) expressed by human non-small lung cancer A549 cells by its EGF ligand. As shown in **Figure 2D**, Cetuximab, Cet-T2m and Cet-T22d35 all inhibited EGFR phosphorylation to a similar extent at various doses, relative to the EGF control, whereas T22d35 alone had no effect on the EGF-induced receptor phosphorylation. These results confirm that fusion of the C-terminal T2m or T22d35 modules to Cetuximab does not interfere with the antibody’s ability to block EGF-induced EGFR phosphorylation.

#### Inhibition of epithelial to mesenchymal transition

It is known that A549 cells exposed to a combination of EGF+TGF-β1 undergo EMT (45), a phenomenon that is characterized by these cells transitioning from an epithelial-like ‘cobble-stone’ morphology to that of a more elongated mesenchymal morphology (**Figure 2E**). This transition is accompanied by changes in the cell-surface expression levels of the adherens junction proteins such as E- and N-cadherin, with E-cadherin levels being down- and N-cadherin levels being up-regulated. We thus assessed the cell surface expression of both E- and N-cadherin by flow cytometry in A549 cells after a 3-day exposure to EGF+TGF-β1 in the presence or absence of Cet-T22d35, Cetuximab+T22d35, and Cetuximab and T22d35 alone. **Figure 2F** clearly shows that Cet-T22d35 significantly inhibits the up- and down-regulation of respectively N-cadherin and E-cadherin, which is better than Cetuximab or T22d35 alone, or even the Cetuximab+T22d35 combination. These results thus demonstrate the superior EMT neutralization potency of the Cet-T22d35 fusion.

#### Inhibition of autocrine EGFR signaling

Disruption of the autocrine EGFR signaling cascade by Cetuximab has been shown to result in varying degrees of cytotoxicity in EGFR-expressing cells (46). To evaluate whether Cet-T22d35 retained this function, we compared the cytotoxicity induced by Cet-T22d35 to that of Cetuximab or T22d35 alone in MDA-MB-468 human breast cancer and HaCaT immortalized human keratinocyte cells. It is known that both cell lines exhibited significant Cetuximab cytotoxicity (47) due to their intrinsic dependence on the EGF signaling cascade for growth. The dose-response curves of Cetuximab, Cet-T22d35 and T22d35 of the HaCaT (**Figure 2G** **, left panel)** and MDA-MD-468 (**Figure 2G** **, right panel)** cell lines show a similar cytotoxic response to both Cetuximab and Cet-T22d35 with calculated IC_50_ values (**Table 4**) in the 0.2-1.4 nM range, while T22d35 elicited no cytotoxic effects. These results further confirm that fusing a TβRII-ED to Cetuximab, and likely other antibody s, does not interfere with the function of antibody itself (https://en.wikipedia.org/wiki/Cetuximab).

**Table 4:** Evaluation of IC_50_ for Cet-T22d35 induced cytotoxicity in MDA-MB-468 and HaCat cells compared to parental Cetuximab and non-fused T22d35.

	IC_50_ (nM)	
	MDA-MB-468	HaCaT
Cetuximab	0.50	0.33
Cetuximab-T22d35	1.42	0.22
T22d35	ND	ND

ND, not detected.

IC_50_ values were calculated using the 4-PL algorithm ((log (inhibitor) vs. response – variable slope (four parameters)) in Graphpad Prism.

#### Pharmacokinetics

To determine whether the Cet-T2m remains intact in circulation, we carried out a PK study in normal, healthy BALB/c mice. Animals were injected with a single dose of Cet-T2m, and the collected serum (15 min to 168 h post-injection) was analyzed by LC-MS/MS MRM using peptides specific for the Fc fragment of Cetuximab (‘ALP’ peptide) and the fused T2m (‘LPY’ peptide). **Table 5** shows that the calculated serum concentrations (Beta_hI) for both peptides are very similar, indicating that the Cet-T2m fusion remains intact over time in circulation, with a relatively long circulating half-life of at least 100 h, which is very similar to the mean serum half-life of ~114 h reported for Cetuximab (https://en.wikipedia.org/wiki/Cetuximab).

**Table 5:** Pharmacokinetic (PK) data for the bifunctional Cet-T2m fusion.

PK parameter	Unit	ALP peptide (Fc)		LPY peptide (TβRII-ED)	
		Estimate	CV%	Estimate	CV%
Alpha_hI	hr	2.1	5.94	3.07	11.54
Beta_hI	**hr**	**99.04**	11.48	**119.84**	19.04
AUC	μg·hr/mL	20142.9	7.39	27455.2	18.81
Cmax	μg/mL	275.48	11.35	309.05	1.79

Serum half-live values (Beta_hI) for the Fc fragment (‘ALP’ peptide) and T2m domain (‘LPY’ peptide) in the Cet-T2m fusion are shown in bold red.

### Blood-brain barrier crossing FC5-Fc-TβRII-ED fusions

**Figure 3:**
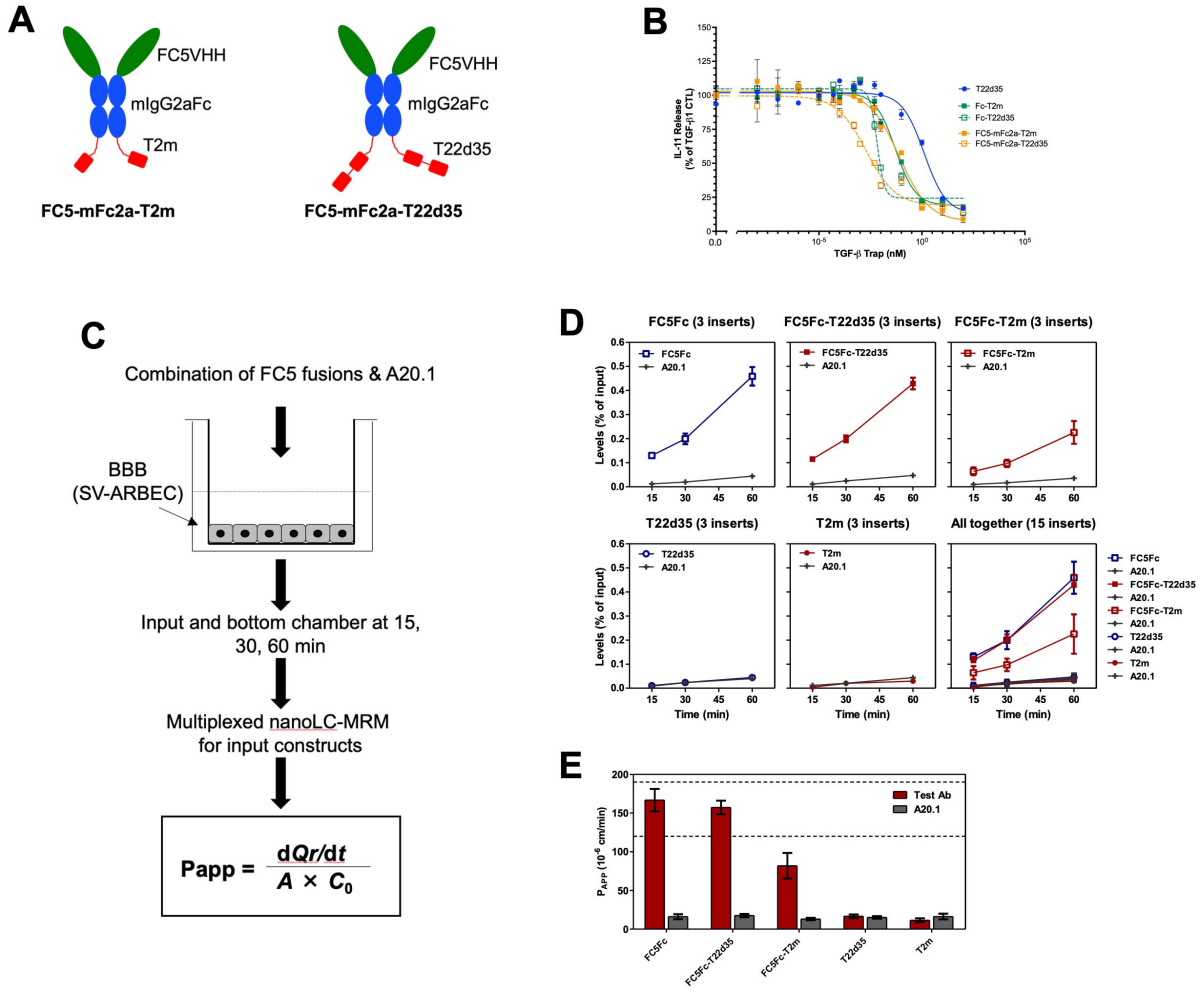
Design and *in vitro* functional evaluation of FC5V_H_H antibody Fc-fused TGF-β traps. **(A)** Schematic representation of the bifunctional FC5V_H_H Fc-fused T2m (left, FC5-Fc-T2m) and T22d35 (right, FC5-Fc-T22d35) constructs (red, TβRII-ED; blue, murine IgG2a Fc fragment; green, FC5V_H_H). **(B)** Assessment of the TGF-β1 neutralization potency in an A549 IL-11 release assay by the FC5-mFc2a-T2m (solid orange squares) and FC5-Fc-T22d35 (open orange squares), compared to the monofunctional T22d35 single chain (blue squares), Fc-T2m (solid green squares) and Fc-T22d35 (open green squares) traps using MSD Mesoscale platform. Graphs show the released IL-11 in the presence of the indicated Trap fusions as the % of IL-11 released by the TGF-β1 control +/- SD. IC_50_ values (see **Table 6**) were calculated using Graphpad Prism (4-PL algorithm ((log (inhibitor) vs. response – variable slope (four parameters)). **(C)** Schematic representation of the *in vitro* Blood Brain Barrier (BBB) assay (for details see text). **(D)** Bifunctional FC5-Fc-T2m and FC5-Fc-T22d35 fusions were assessed for their ability to cross a rat SV-ARBEC barrier in an *in vitro* BBB assay over time. The efficiency of BBB permeability was calculated by nanoLC-MRS and expressed as (output bottom chamber/input top chamber) x100%. **(E)** The apparent permeability coefficient (P_app_) value, which is a measure of transport across the BBB, was calculated at t=60 min for the FC5-Fc-T2m and FC5-Fc-T222d35 fusions and compared to monofunctional FC5-Fc, non-fused T22d35 and T2m traps, and the non-crossing A20.1 control. Bar graphs represent a representative triplicate experiment +/- SD that was repeated at least 3 times.

In addition to the antibody fusions, we also linked the T2m and T22d35 C-terminally to the Fc fragment of a well characterized BBB-penetrating single-domain antibody FC5V_H_H-Fc construct (**Supplementary Table S5**). FC5V_H_H binds TMEM30a and allows the FC5V_H_H-Fc to undergo receptor mediated transcytosis across the BBB (31). Fusion of the T2m or T22d35 to FC5V_H_H-Fc would thus facilitate transport of FC5-Fc_T2m and FC5-Fc-T22d35) across the BBB and allow for targeting TGFβ in the brain. FC5-Fc-T2m and FC5-Fc-T22d35 (**Figure 3A**), were produced and purified **(see** **Supplementary Figure S1GH**), and then evaluated for their ability to neutralize TGF-β1, and cross the BBB using an *in vitro* SV-ARBEC BBB trans-well model system.

#### TGF-β1 neutralization

The TGF-β1 neutralization potency of both the FC5-Fc-T2m and FC5-Fc-T22d35 was compared to that of the Fc-T2m, Fc-T22d35 and the non-Fc-fused T22d35 single-chain trap in the described A549 IL-11 release assay. The data presented (**Figure 3B**), shows that efficient TGF-β1 neutralization was conserved in the both fusions (**Table 6**), with K_D_ values of 5.688 pM for FC5-Fc-T2m and 2.351 pM for FC5-Fc-T22d35. These values are in agreement with those for the other trap fusions, and confirms that TGF-β1 neutralization can be achieved to a much higher degree with a T22d35 fusions compared to a Fc-T2m fusion or non-fused T22d35.

**Table 6:** Evaluation of the bifunctional FC5-Fc-T2m and FC5-Fc-T22d35 traps in the A549 IL-11 release assay.

Bifunctional FC5-fused traps	IC_50_ (nM)
	TGF-β1
FC5-Fc-T2m	0.005688
FC5-Fc-T22d35	0.002351

IC_50_ values were calculated using a 4-PL algorithm ((log (inhibitor) vs. response – variable slope (four parameters)) in Graphpad Prism.

#### Blood-brain barrier crossing

We then assessed the ability of the two TGF-β trap fusion proteins, FC5-Fc-T2m and FC5-Fc-T22d35, to undergo receptor-mediated transcytosis across the BBB *in vitro*. Specifically, we assessed the efficiency and apparent permeability (P_app_) of FC5-Fc-T22d35 and FC5-Fc-T2m in comparison to control antibodies including FC5-Fc, T22d35, T2m, and the non-transcytosing A20.1 antibody, raised against *Clostridium difficile* toxin A with no know mammalian receptor (48). The BBB transcytosis studies were performed using a trans-well setup (49) (**Figure 3C**), wherein the SV-ARBEC cells were seeded on collagen I-coated semi-permeable inserts (42) with the test antibodies being added to the top apical (input) and collected from the top and bottom basolateral (output) chambers at defined time points (15, 30, 45 and 60 min). The transcytosis efficiency and P_app_ were quantified by highly sensitive multiplexed nanoLC-SRM (31, 43). The transcytosis efficiency was calculated as (output/input) × 100%, representing the percentage of the applied compound that successfully traversed the SV-ARBEC monolayer into the basolateral compartment over the experimental time frame. The transcytosis efficiency for FC5-Fc-T22d35 (top middle) was very similar to the FC5-Fc control (top left) (**Figure 3D**). In contrast, the transcytosis of the FC5-FcT2m (top right) was lower, but much higher than those of T2m (bottom middle) and T22d35 (bottom left) alone which showed negligible BBB permeability, similar to the non-crossing A20.1 control fusion (**Figure 3D**). These results are supported by the P_app_ values at 60 min, which demonstrate that the permeability of FC5-Fc-T22d35 and the FC5-Fc control were approximately 50% higher than those observed for FC5-Fc-T2m (**Figure 3E**). Nevertheless, the levels of FC5-Fc-T2m detected were still ~4-fold higher than that of the negative controls (T2m, T22d35, and A20.1). Overall, this data thus shows that the FC5 V_H_H retains its functional capacity to mediate receptor-dependent transcytosis when fused to TGF-β traps, enabling efficient transport of FC5-Fc-T22d35 across the BBB.

### Bone-homing D10-Fc-T2m fusions

**Figure 4:**
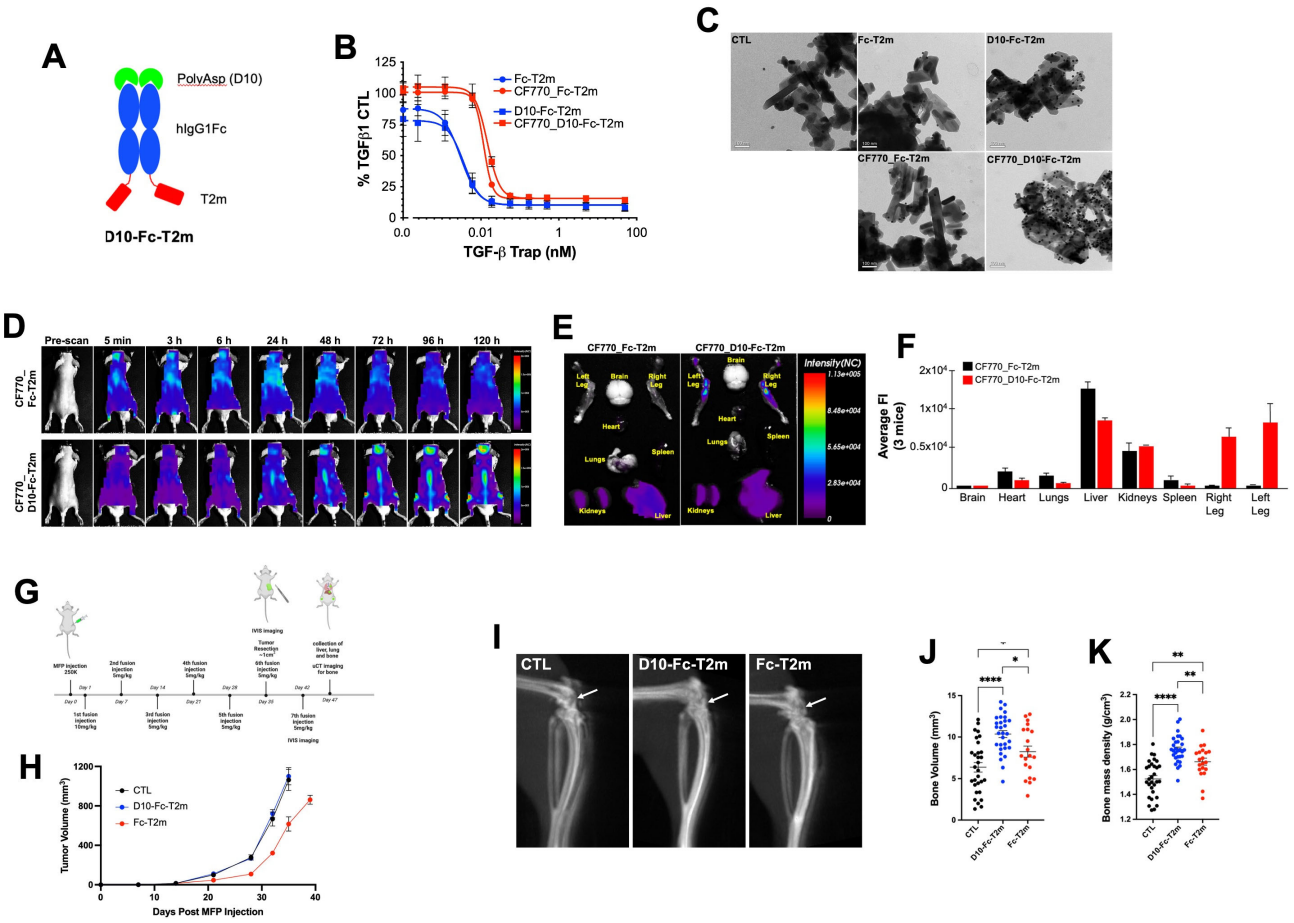
Design and *in vitro* and *in vivo* functional evaluation of D10 Fc-fused bifunctional TGF-β traps. **(A)** Schematic representation of the bifunctional D10 Fc-fused T2m (D10-Fc-T2m) fusion (red, TβRII-ED; blue, human IgG1 Fc fragment; green, poly-aspartate (D10)). **(B)** Evaluation of the TGF-β1 neutralization by the unlabeled (blue) CF770 labelled (red) D10-Fc-T2m (solid squares) or Fc-T2m (solid circles) in an A549 IL-11 release assay using the MSD Mesoscale platform. Graphs represent a representative triplicate experiment +/- SD that was repeated at least 3 times. IC_50_ values were calculated (**Table 7**) using Graphpad Prism (4-PL algorithm ((log (inhibitor) vs. response – variable slope (four parameters)). **(C)** TEM photographs showing the specific binding of D10-Fc-T2m (top right) and CF770_D10-Fc-T2m (bottom right), but not Fc-T2m (top middle) or CF770_Fc-T2m (bottom middle) to hydroxyapatite crystals and detected by a gold particle labelled anti-Fc antibody (black dots). Hydroxyapatite crystals incubated with gold particle labelled anti-Fc antibody alone was used as control (top left). **(D)** Representative whole mouse images (n=3) of the distribution of CF770-labeled Fc-T2m (top) and D10-Fc-T2m (bottom) fusions, followed 5 min to 120h post-injection, were obtained using the eXplore Optix pre-clinical imager MX3. The data demonstrates that presence of a poly-aspartate (D10) motif targets and retains the CF770_D10-Fc-T2m, but not the CF770_Fc-T2m, in the vertebrae, and cranial and leg bones of the mouse. **(E)** *Ex vivo* images of dissected organs and bones (t=120h post injection) showing that CF770_D10-Fc-T2m and CF770_Fc-T2m can be found in the kidneys and liver, but that only CF770_D10-Fc-T2m can be detected in the right and left leg bone. **(F)** Quantitation of the average fluorescence intensities (eXplore Optix Optiview analysis software 3.02) of the *ex vivo* organs (t=120h post-injection) confirm the bone-specific accumulation of the D10-Fc-T2m (red bars), but not the Fc-T2m fusion (black bars). **(G)** Cartoon outlying the experimental *in vivo* approach that was used for the D10-Fc-T2m assessment. **(H)** Graph depicting the increase in volume (mm^3^) of primary MDA-MB-231 TR ZsGreen+ mammary tumors for up to 40 days post-implantation in animals treated with D10-Fc-T2m, Fc-T2m and PBS (CTL). X-ray microcomputed tomography (µCT) images of mouse leg bones (20 days post-tumor resection) showing the impairment in osteolytic lesion formation **(I,** arrow), leading to higher bone volumes **(J)** and bone mass densities **(K)** in D10-Fc-T2m, and to a lesser extent Fc-T2m treated animals, compared to PBS injected animals.

We also investigated whether the addition of a 10 amino-acid-long poly-aspartate bone-localization motif (D10) (32, 38) at the N-terminus of the Fc-fused TβRII-ED trap would allow targeting of a TGF-β neutralizing moiety specifically to the bone (D10-Fc-T2m**;** **Figure 4A**). The D10-Fc-T2m was expressed and purified (see **Supplementary Figure S1I**), and compared to the Fc-T2m fusion, which lacks the D10 sequence, in an *in vitro* and *in vivo* setting. To facilitate monitoring of bone targeting and retention *in vivo*, we also labelled the D10-Fc-T2m with the CF770 near-infrared dye.

#### TGF-β1 neutralization

Fusion of the poly-aspartate D10 motif to the N-terminus of the Fc-T2m construct did not impact TGF-β1 neutralization (**Figure 4B**, blue graphs), showing an IC_50_ value that is very similar to the Fc-T2m fusion (**Table 7**; 3.794 pM *versus* 2.895 pM, respectively). However, CF770 labelling of the Fc-T2m and D10-Fc-T2m fusion reduced TGF-β1 neutralization by ~4-fold (**Figure 4B**, red graphs), which was not entirely surprising. The TβRII-ED/TGF-β binding interface contains several lysine residues, and although our CF770 conjugation strategy was aimed at foremost labeling lysines in the Fc fragment, those in the receptor/ligand interface were likely also labeled, thereby compromising TGF-β neutralization to some extent. It should be noted that unlabeled D10-Fc-T2m and Fc-T2m were used for both the PK and *in vivo* efficacy studies (described in following sections), hence the TGF-β neutralizing capacity of the constructs used in those studies is thus not compromised.

**Table 7:** Evaluation of unlabeled and CF770-labeled bifunctional D10-Fc-T2m and Fc-T2m traps in the A549 IL-11 release assay.

Bifunctional D10-fused traps	IC_50_ (nM)
	TGF-β1
Fc-T2m	0.002895
CF770_Fc-T2m	0.01172
D10-Fc-T2m	0.003794
CF770_D10-Fc-T2m	0.01453

IC_50_ values were calculated using a 4-PL algorithm ((log (inhibitor) vs. response – variable slope (four parameters)) in Graphpad Prism.

#### Binding to hydroxyapatite

The bone-binding capacity of the D10-fusions was confirmed by their direct binding to hydroxyapatite, an inorganic mineral present in both human bone and teeth. Transmission electron microscopic images (TEM**;** **Figure 4C**) show an abundance of 14 nm gold-particle-protein A conjugate along the surface (black dots) of the hydroxyapatite crystals that were incubated the D10-Fc-T2m and CF770_D10-Fc-T2m. Only few gold particles were associated with the hydroxyapatite crystals that were incubated with Fc-T2m and CF770_Fc-T2m, which lack the D10 sequence, and the hydroxyapatite that was incubated with gold-particle-protein A alone (conjugate negative control). These data indicates that the D10 moiety retains its bone-binding characteristics in the context of the trap fusions.

#### *In vivo* imaging studies

To evaluate the ability of the D10 fusions to home to and be retained in the bone we injected healthy mice with CF770_Fc-T2m or CF770_D10-Fc-T2m. **Figure 4D** shows representative images of mice (n=3) injected with a single dose of either CF770_Fc-T2m (top) or CF770_D10-Fc-T2m (bottom). These results demonstrate that addition of the D10 peptide to the N-terminus of the Fc-T2m significantly enhances bone localization and retention, and images taken between 5 min to 120 h post-injection show a clear accumulation of CF770_D10-Fc-T2m, but not CF770_Fc-T2m, in the skull bones, hind legs and vertebrae. *Ex vivo* imaging of the brain, heart, lungs, liver, kidneys, spleen and hind legs 120h post-injection, and the average Fluorescent Intensity (FI) for the indicated organs further confirms the specific accumulation of the D10-Fc-T2m but not the Fc-T2m in the bones of the hindlimbs (**Figures 4E, F**). The fluorescent signals observed in the kidneys and liver are similar for both fusions, indicating that accumulation in these organs is not driven, nor affected by the presence of the D10 sequence.

#### Pharmacokinetics

To determine the serum half-life of the Fc-T2m and D10-Fc-T2m trap fusions, we carried out an *in vivo* PK study in normal, healthy BALB/c mice. Mice were injected with a single dose of Fc-T2m or D10-Fc-T2m and the collected serum samples (15 min to 168 h post-injection) were analyzed by LC-MS/MS MRM using the method described. Our results indicate that the D10-Fc-T2m has a much shorter serum half-live than Fc-T2m (~50 h and ~130 h, respectively; **Table 8**), the latter of which is similar to the value calculated for Cet-T2m (**Table 5**; ~120 h). The shorter serum half-life of D10-Fc-T2m is likely attributed to the presence of the poly-aspartate motif causing its accumulation and retention over time in the mouse bones (**Figures 4D-F**).

**Table 8:** Comparison of the pharmacokinetic (PK) data of the bifunctional D10-Fc-T2m and monofunctional Fc-T2m fusions.

PK parameter	Unit	Fc-T2m				D10-Fc-T2m			
		ALP peptide (Fc)		LPY peptide (TβRII-ED)		ALP peptide (Fc)		LPY peptide (TβRII-ED)	
		Estimate	CV%	Estimate	CV%	Estimate	CV%	Estimate	CV%
Alpha_hI	hr	2.85	18.69	2.84	15.67	3.61	62.41	4.08	85.20
Beta_hI	**hr**	**132.86**	8.08	**116.89**	8.50	**51.64**	13.60	**53.22**	14.51
AUC	μg·hr/mL	27913.60	6.69	27107.50	6.43	12794.8	9.07	13182.10	10.41
Cmax	μg/mL	278.70	2.87	314.07	3.03	303.54	53.30	301.21	60.79

Serum half-live values (Beta_hI) for the Fc fragment (‘ALP’ peptide) and TβRII-ED (‘LPY’ peptide) in the fusions are shown in bold red.

#### *In vivo* efficacy studies

To further investigate whether the D10-Fc-T2m fusion could affect the formation of breast cancer bone metastases, we used MDA-MB-231 human breast cancer cells that were implanted in the mammary fat pad (MFP) of NOD SCID gamma (NSG) mice as an *in vivo* model for spontaneous bone metastasis. Starting one day post tumor cell implantation, Fc-T2m or D10-Fc-T2m trap fusions were administered intravenously (iv, tail vein) each week, using PBS as a negative control (**Figure 4G**). The growth of primary mammary tumors was similar for the PBS and D10-Fc-T2m infused mice; whereas Fc-T2m infused mice exhibited a slight delay in tumor growth (**Figure 4H**). This is likely due to the fact the effective circulating Fc-T2m levels are higher than the D10-Fc-T2m (due to the fact that D10-Fc-T2m accumulates in the bone), which correlates with its PK profile (**Table 8**). Tumors were resected once mammary tumors reached approximately 1 cm^3^ in size, and bone metastatic lesions were allowed to form until mice exhibited paralysis. Using μCT imaging, we quantified the degree of bone destruction that could be observed in the proximal tibia of the indicated mice. The osteolytic lesions that are formed in mice receiving either the Fc-T2m or D10-Fc-T2m trap fusions injected mice were significantly smaller and less destructive when compared to the metastases formed in the PBS injected mice (**Figure 4I**). However, the D10-Fc-T2m fusion was significantly more effective in impairing the formation of osteolytic bone lesions than the Fc-T2m trap, which is evident by the higher bone volumes and bone mass density in D10-Fc-T2m treated animals (**Figure 4J, K**). Overall, these results thus suggest that the D10-Fc-T2m trap fusion, which localizes and accumulates to the bone surface, can effectively impair the formation of osteolytic bone metastatic lesions.

### Fc-fused trap activation of the immune system

**Figure 5:**
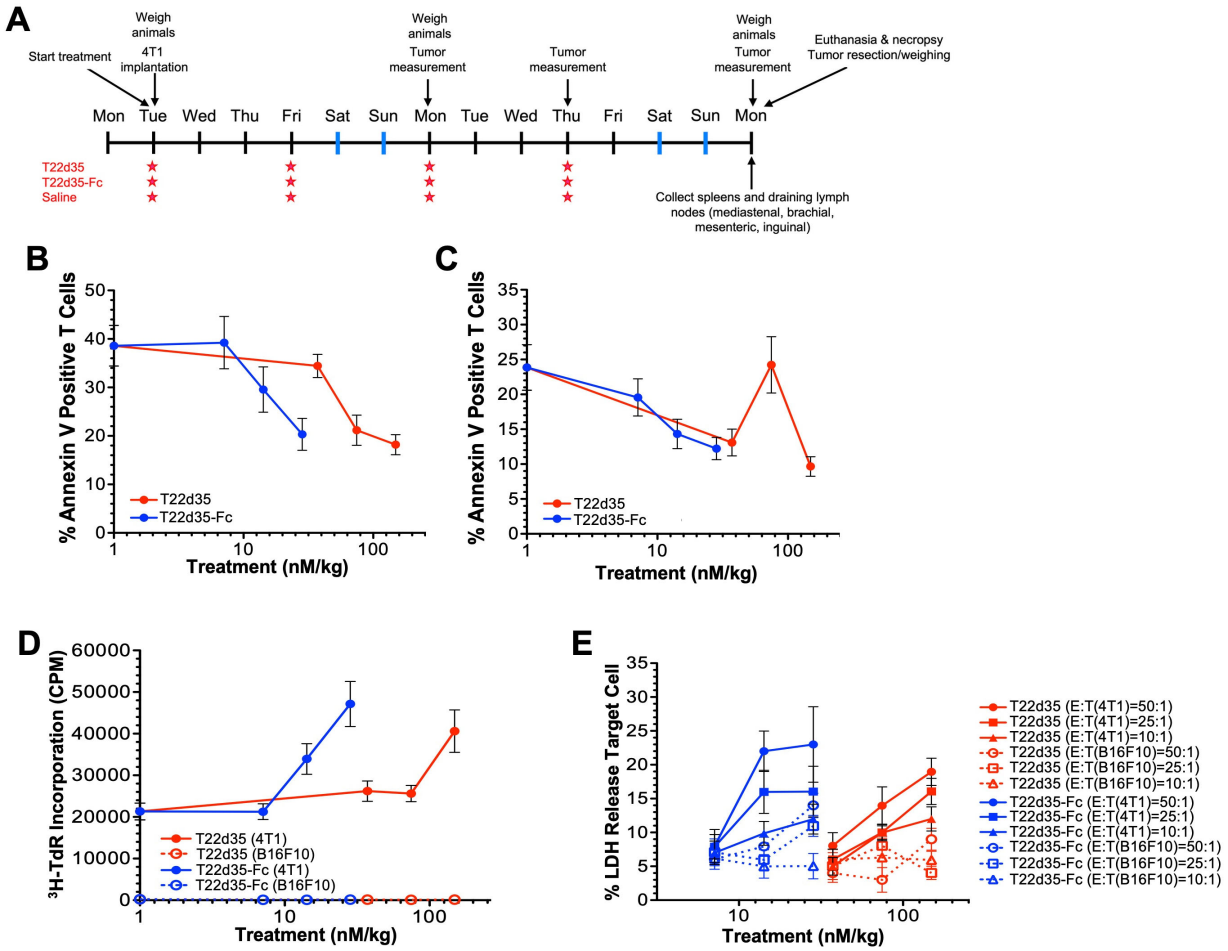
T22d35-Fc treatment potently activates the immune system in 4T1 tumor bearing animals. **(A)** Outline of the *in vivo* study. Briefly, 4T1 cells were implanted subcutaneously with treatment starting on the same day. Animals were treated 2x/week (10 mg/kg) for 2 weeks after which animals were euthanized, and spleen and draining lymphnodes were harvested for T-cell isolation. *Ex vivo* evaluation of isolated CD4^+^ and CD8^+^ T cells from 4T1 tumor bearing animals treated with T22d35-Fc (blue) are less likely to undergo apoptosis **(B, C)** and proliferate **(D)** then those isolated from animals treated with non-fused T22d35 (red). **(E)** In addition, the ability of these isolated T cells (E; effector cell) to lyse 4T1 cells (T; target cell) *ex vivo* at various E:T ratios are superior to that of T cells isolated from animals treated with non-fused T22d35. Using the B16F10 mouse melanoma tumor cell line (open symbols) instead of 4T1 cells (closed symbols) as target cell shows that the T cell response is 4T1 specific.

*In vivo* data evaluation of the immune response (**Figure 5A**) shows that the T22d35-Fc trap effectively activates the immune system in 4T1 tumor bearing immunocompetent BALB/c mice. *Ex vivo* evaluation of CD4^+^ (**Figure 5B**) and CD8^+^ (**Figure 5C**) T cells isolated from T22d35-Fc treated 4T1 tumor bearing animals are less likely to undergo apoptosis and proliferate better (**Figure 5D**). Our data also shows that T cells isolated from animals treated with the T22d35-Fc more potently lyse 4T1 cells *ex vivo* compared to T cells isolated from non-fused T22d35 treated animals (**Figure 5E**). In addition, the lack of response in B16F10 mouse melanoma tumor cells shows that the T cell response is 4T1 specific.

## Discussion

Members of the TGF-β superfamily have been shown to play a key role in the regulation of normal physiological processes by activating intricate canonical and non-canonical signaling pathways (50), that are often de-regulated in pathologies such as cancer (51, 52). When TGF-β acts as a tumor promotor, it suppresses both the innate and adaptive immune systems and enhances tumor cell proliferation, migration and invasion, which collectively impact drug resistance and tumor escape, and undermine a clinical response to anticancer therapy (3). Its broad expression pattern and dual role as both a tumor suppressor and tumor promotor has made targeting TGF-β a challenge. Various approaches have been used to neutralize TGF-β signaling, which includes small molecule TGF-β receptor kinase inhibitors, antisense oligonucleotides and vaccines, and monoclonal antibodies (53–56).

Another method to neutralize TGF-β is the use of soluble forms of the TGF-β Type II Receptor Ecto Domain (TβRII-ED) as a ligand trap. By using a novel protein engineering design strategy, we previously generated a single-chain, bivalent TGF-β Type II receptor ectodomain trap (T22d35). This trap potently neutralized TGF-β1 and -β3, and not -β2, but had a very short circulating half-life of less than 1h (28, 35, 36). Extension of its serum half-life can be achieved by linking short-lived proteins such as the trap to the C-terminus of an antibody.

We demonstrated that by using antibody-based drugs like Cetuximab, Herceptin, Avastin and Synagis, bifunctional fusion proteins cold be generated that retained both their original antibody function and the ability to neutralize TGF-β. Others have successfully used the same approach, for example, Binatrafusp alfa (57) and SHR-1701 (58) are anti-PD-L1 C-terminally fused TβRII-ED bifunctional fusion proteins, that have both been evaluated in clinical trials either as mono- or combination therapy for the treatment of several types of cancer (59–65). Several alternative antibody fusions have also been designed recently, for example YM101/BiTP (66) is a hybrid bifunctional antibody developed through the Check-BODY™ technology platform, which fuses the TβRII-ED to the antibody light chain. In addition, Biofusion is developing Ficerafusp alfa [targeting EGFR and TGF-β; (67, 68)] which fused a TβRII-ED to the N-terminus of the light chain of Cetuximab IgG via a flexible (G_4_S)_3_ linker. Although antibody-fused TGF-β trap fusions are attractive from a manufacturing cost point-of-view, caution should be exercised in their use in terms of treatment timing (i.e., disease stage, tumor type, and its use in combination therapies), given TGF-β‘s dual role as a tumor suppressor and tumor promotor.

An alternative to the full-size antibody fusions is to link the TβRII-ED-based trap to only the Fc fragment of an antibody, an approach that adds more flexibility in terms of treatment timing. To this end, we engineered a series of N- and C-terminally Fc-fused traps and evaluated the use of the four human IgG isotypes Fc regions. We used different sequences to link the trap to the Fc fragment and modified the Fc hinge regions to avoid aggregation and potential immunogenicity issues. In this manner, we identified the T22d35-hIgG2Fc(CC)ΔK N-terminal trap fusion (indicated in bold in **Supplementary Table S4**) as our lead in which good manufacturability is combined with potent TGF-β neutralization. *In vivo*, this trap was also shown to stimulate a “T-cell-inflamed” tumor state by 1) promoting the infiltration of T cells **into** the tumor environment, 2) preventing T cells to undergo apoptosis, 3) inducing T cell proliferation, and 4) enabling T cells to efficiently and specifically lyse tumor cells (69). On the basis of its favorable manufacturing and functional characteristics this trap fusion was further developed under the name AVID200 by both Forbius and Bristol Myers Squibb (69), and was assessed in several clinical trials (NCT03834662, NCT03831438, NCT03895112), where it was reported to be an effective and well-tolerated therapeutic in oncology, and for the treatment of myelofibrosis (70). It should be noted that a direct comparison of Merck’s Binatrafusp alfa to AVID200 in an A549 IL-11 release assay showed the latter to be slightly more potent in neutralizing TGF-β1 and -β3 (**Supplementary Figures S3A-C**).

There are other TGF-β targeting Fc-fused traps in the literature, for example Takahashi et al. (71) reported on a TβRI-TβRII-Fc-fusion that neutralizes all TGF-β isoforms. However, the advantage of our T22d35-Fc-fusion is that it is ~1500 times more selective for TGF-β1 and -β3 compared to TGF-β2. While TGF-β2 is a positive regulator of hematopoiesis and normal cardiac function, and TGF-β1 and -β3 are negative regulators of hematopoiesis, thus makes the T22d35-Fc-fusion a very attractive therapeutic modality for the treatment of myelodysplastic syndrome (MDS) associated anemia.

It is often desirable to guide the TGF-β neutralization to a specific organ to achieve a potent local effect and limit exposure to healthy tissues. To this end, we designed and generated bifunctional Fc-fused TGF-β traps by further fusing them to homing moieties. Importantly, this approach demonstrated the versatility of our Fc-fused TGF-β traps while maintaining good functional and manufacturing attributes. In a first set of bifunctional trap examples, we linked a single domain antibody with blood-brain-barrier (BBB) crossing ability (FC5V_H_H) to the N-terminus of Fc-TβRII-ED based traps. In a second example of bifunctional traps, we linked a poly-aspartate bone-homing peptide (D10) to the N-terminus of Fc-TβRII-ED based traps. The bifunctional traps incorporating the FC5 module retained the crossing of an *in vitro* BBB model composed of immortalized rat brain endothelial cells (SV-ARBECs) demonstrated for FC5 (31, 37). Since Lessard et al. showed that an FC5-Fc-fusion is capable of delivering therapeutic payloads into the CNS of rodents and dogs (72), and it is expected that our FC5-Fc trap fusions will also be successfully shuttled across the BBB. Given that the expression of TGF-β1 and -β2 strongly correlates to poor survival in patients with glioblastoma (73) and the notion that our engineered bifunctional FC5-Fc-fusions neutralize TGF-β1 and TGF-β2 (albeit to a lesser extent) underscores the therapeutic potential of these molecules.

We also developed bifunctional Fc-fused TGF-β traps that contain a poly-aspartate sequence (D10). This motif has a strong affinity for hydroxyapatite, which is the main mineral component of bone. Bone is also very rich in TGF-β and other stored growth factors, creating an ideal environment in which tumor cells can thrive (74, 75). Nonetheless, clinical trials using TGF-β inhibitors for the treatment of bone metastasis have yielded limited survival benefits and some adverse effects likely arise since TGF-β is so broadly expressed throughout the body. Using a breast cancer metastasis *in vivo* mouse model, we showed that our bifunctional D10-Fc-TβRII-ED based trap, but not the version lacking the D10 motif, homes to and accumulates in the bone, leading to a reduction in the formation of osteolytic bone lesions. Tian et al. inserted six aspartate long peptides at various positions into either the heavy or light chain of Trastuzumab and showed that an antibody drug conjugate (ADC) version of this engineered antibody can inhibit breast cancer primary growth and metastases (76). Nonetheless, caution should be exercised using this approach, as too many aspartate motifs prevent the release of the ADC causing a suboptimal ADC activity against bone metastases. Since TGF-β regulates a feed-forward cycle of tumor growth in bone that favors of osteolysis (77), our bifunctional D10-Fc-TβRII-ED based trap has the advantage that it homes and retains the TβRII-ED with high affinity in the bone matrix where in can function as a constant TGF-β neutralizer. This approach may thus alleviate some of the undesirable side effects observed when using a systemic approach.

Decades of research have demonstrated the complex role TGF-β plays in the multistep process of cancer metastasis. And although selective pharmacological inhibitors have been used to target TGF-β‘s tumor promoting activities, their promising pre-clinical data has failed to translate to the clinic (78). Blocking TGF-β function alone typically does not kill cancer cells, however thwarting its function can enhance the efficacy of other cancer treatments such as radio-, chemo and especially immune-therapy (79–82).

The study presented here demonstrates that our TGF-β1 and -β3 specific Fc-fused TβRII-ED can be produced and purified at large scale, either as a mono- or bifunctional fusion, while combining potent TGF-β neutralization and targeting specificity with a serum half-life that is comparable to that of a monoclonal antibody. We also showed that TGF-β neutralization can be tweaked by either fusing a single or tandem TβRII-ED molecules to either the N- or C-terminus of an Fc fragment, and that these Fc fusions can be combined with a second therapeutic moiety to deliver bifunctional molecules. These can be engineered in the context of an antibody, or by using a ‘homing sequence’ that allows its targeting to a specific organ or microenvironment. Such fusions thus establish a new strategy for the precision neutralization of TGF-β will allow to transition from traditional only antigen-specific therapies to therapies that are both antigen- and tissue/microenvironment-specific therapies, which can also be used in combination therapies. The multifunctional approach to TGF-β neutralization described in this study has the potential to harness and reduce the side effects observed when systemically targeting TGF-β, thus providing a new avenue for advancing TGF-β targeted therapy toward the clinic.

## Data Availability

The original contributions presented in the study are included in the article/**Supplementary Material**. Further inquiries can be directed to the corresponding author.
