# Supplementary figures and images for: Engineered multifunctional transforming growth factor-β type II receptor ectodomain fusions for oncology applications
Source: Front Oncol. 2025 Oct 27;15:1648779. doi: 10.3389/fonc.2025.1648779 (PMC12597760; doi:10.3389/fonc.2025.1648779)

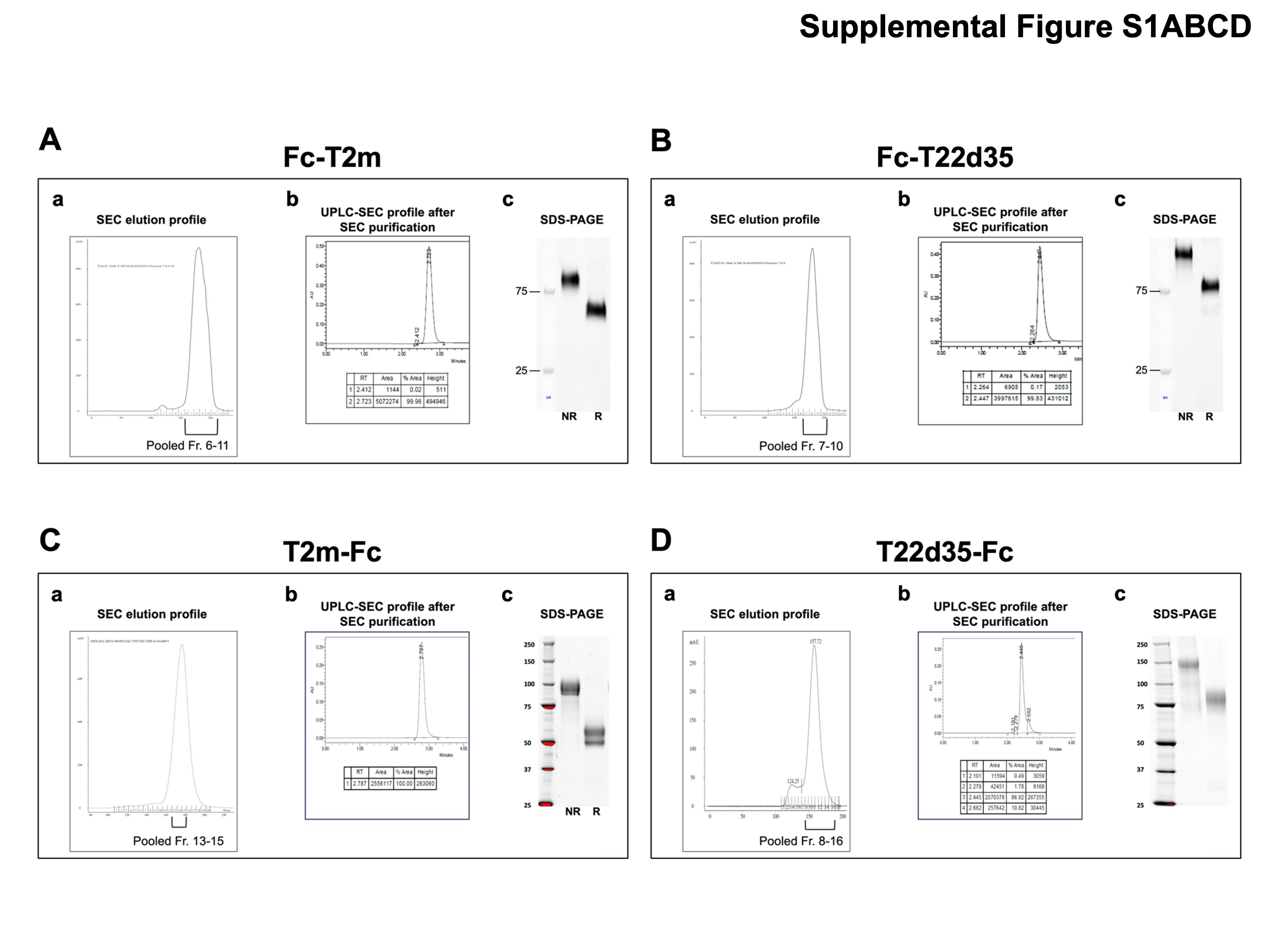

Supplement: Supplementary file 1 [file Image1.tif]

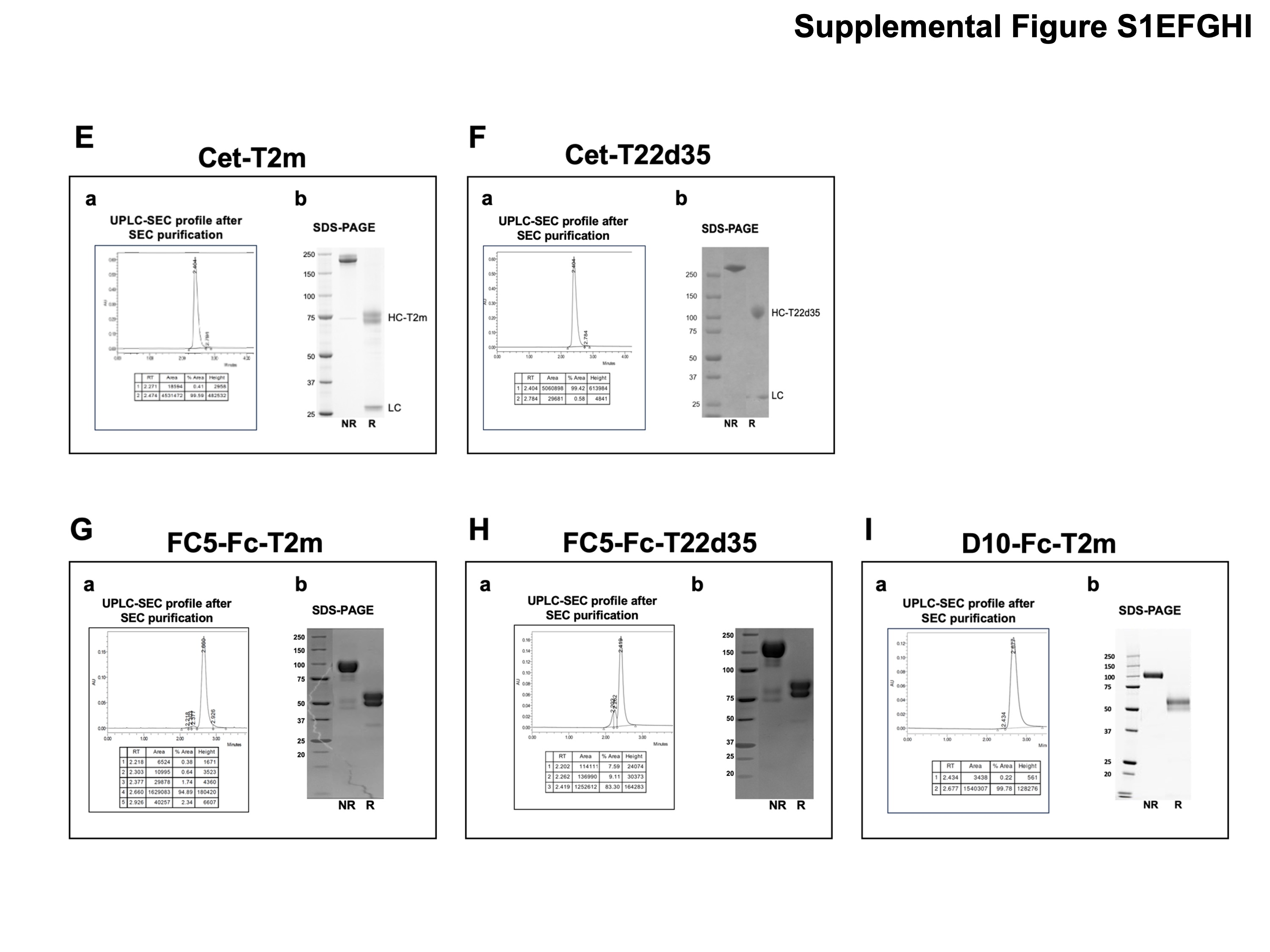

Supplement: Supplementary file 2 [file Image2.tif]

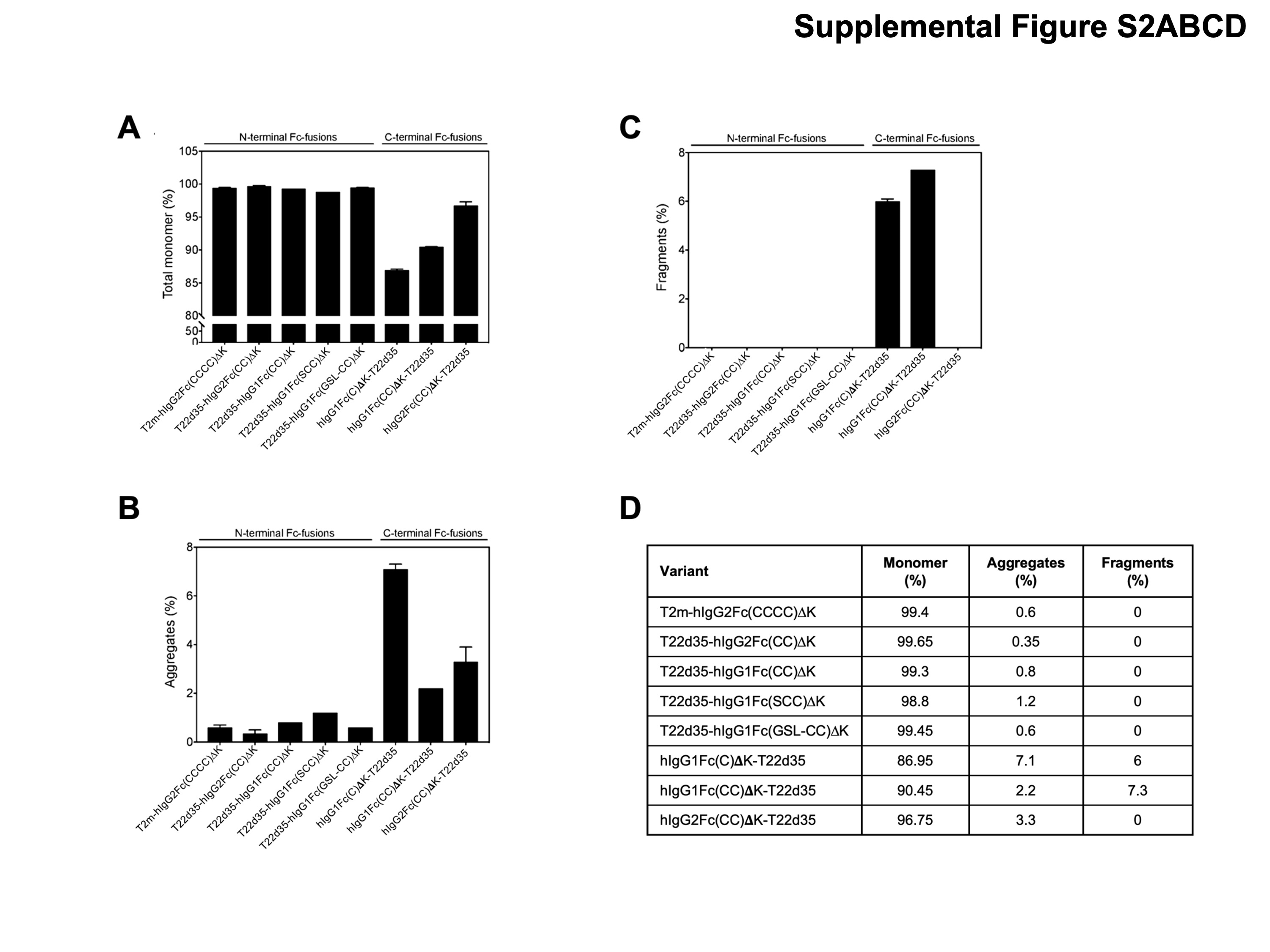

Supplement: Supplementary file 3 [file Image3.tif]

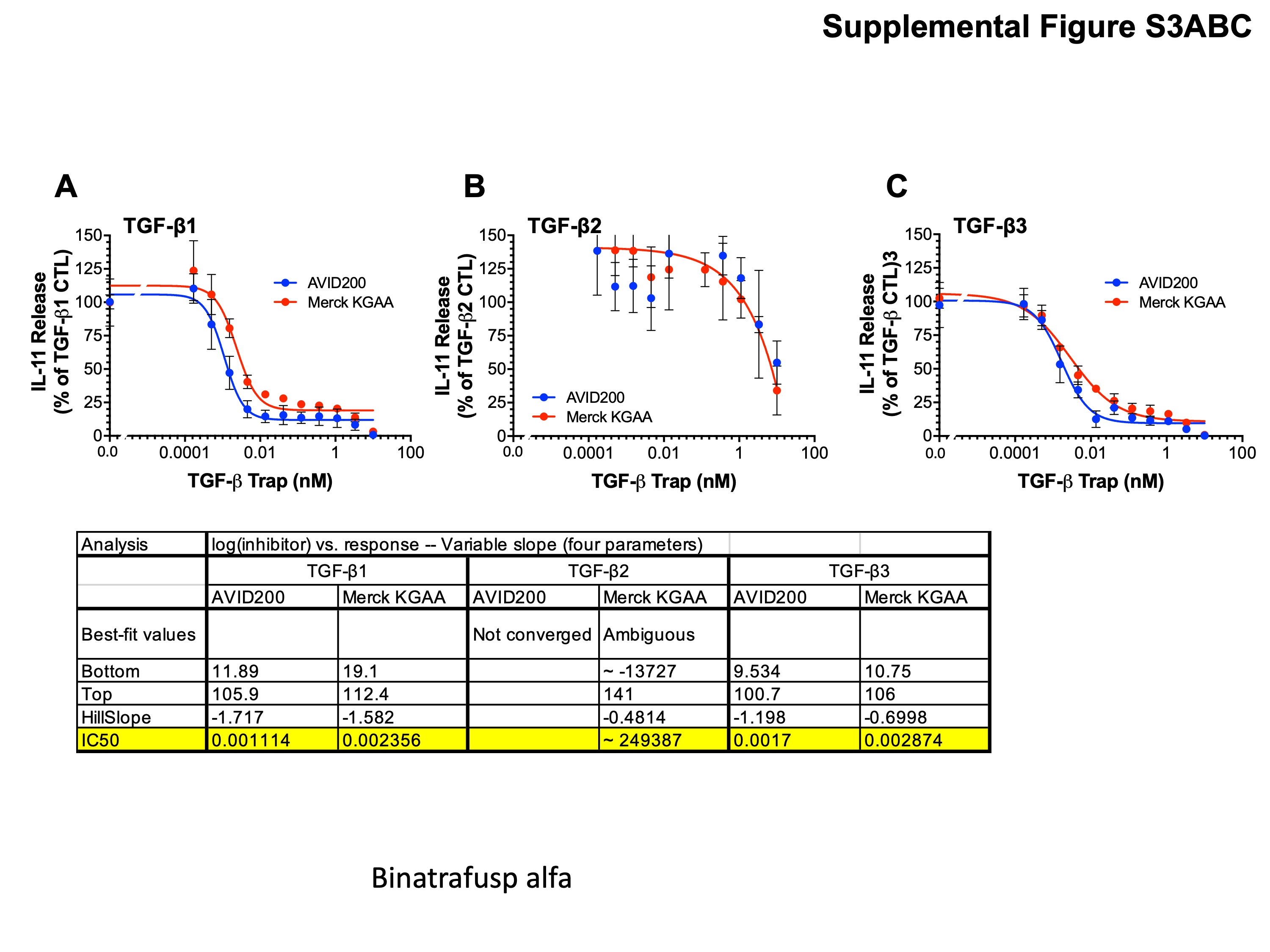

Supplement: Supplementary file 4 [file Image4.tif]
