# Supplementary material for: Engineered multifunctional transforming growth factor-β type II receptor ectodomain fusions for oncology applications
Source: Front Oncol. 2025 Oct 27;15:1648779. doi: 10.3389/fonc.2025.1648779 (PMC12597760; doi:10.3389/fonc.2025.1648779)
Supplement: Supplementary file 5 [file Table1.docx]

**SUPPLEMENTAL TABLES**

**Supplemental Table S1:** Amino acid sequences of the T2m and T22d35 Traps. Natural linker sequences are in underlined.

| **T2m** | IPPHVQKSVNNDMIVTDNNGAVKFPQLCKFCDVRFSTCDNQKSCMSNCSITSICEKPQEVCVAVWRKNDENITLETVCHDPKLPYHDFILEDAASPKCIMKEKKKPGETFFMCSCSSDECNDNIIFSEEYNTSNPD |
| --- | --- |
| **T22d35** | IPPHVQKSVNNDMIVTDNNGAVKFPQLCKFCDVRFSTCDNQKSCMSNCSITSICEKPQEVCVAVWRKNDENITLETVCHDPKLPYHDFILEDAASPKCIMKEKKKPGETFFMCSCSSDECNDNIIFSEEYNTSNPDIPPHVQKSVNNDMIVTDNNGAVKFPQLCKFCDVRFSTCDNQKSCMSNCSITSICEKPQEVCVAVWRKNDENITLETVCHDPKLPYHDFILEDAASPKCIMKEKKKPGETFFMCSCSSDECNDNIIFSEEYNTSNPD |

**Supplemental Table S2:** Amino acid sequences of the ‘headless’ C-terminally hIgG1, hIgG2, hIgG3 and hIgG4 Fc-fused T2m constructs. hIgGFc heavy chain, blue; T2m sequence, black; natural linker sequences, black underlined; Fc hinge region, bold blue; paired cysteine residues, bold red; serine replaced cysteines, bold red underlined.

| **hIgG1Fc(SCC)ΔK-T2m** | **EPKSSDKTHTCPPCPAPE**LLGGPSVFLFPPKPKDTLMISRTPEVTCVVVDVSHEDPEVKFNWYVDGVEVHNAKTKPREEQYNSTYRVVSVLTVLHQDWLNGKEYKCKVSNKALPAPIEKTISKAKGQPREPQVYTLPPSRDELTKNQVSLTCLVKGFYPSDIAVEWESNGQPENNYKTTPPVLDSDGSFFLYSKLTVDKSRWQQGNVFSCSVMHEALHNHYTQKSLSLSPGIPPHVQKSVNNDMIVTDNNGAVKFPQLCKFCDVRFSTCDNQKSCMSNCSITSICEKPQEVCVAVWRKNDENITLETVCHDPKLPYHDFILEDAASPKCIMKEKKKPGETFFMCSCSSDECNDNIIFSEEYNTSNPD |
| --- | --- |
| hIgG1Fc(SSC)ΔK-T2m | **EPKSSDKTHTSPPCPAPE**LLGGPSVFLFPPKPKDTLMISRTPEVTCVVVDVSHEDPEVKFNWYVDGVEVHNAKTKPREEQYNSTYRVVSVLTVLHQDWLNGKEYKCKVSNKALPAPIEKTISKAKGQPREPQVYTLPPSRDELTKNQVSLTCLVKGFYPSDIAVEWESNGQPENNYKTTPPVLDSDGSFFLYSKLTVDKSRWQQGNVFSCSVMHEALHNHYTQKSLSLSPGIPPHVQKSVNNDMIVTDNNGAVKFPQLCKFCDVRFSTCDNQKSCMSNCSITSICEKPQEVCVAVWRKNDENITLETVCHDPKLPYHDFILEDAASPKCIMKEKKKPGETFFMCSCSSDECNDNIIFSEEYNTSNPD |
| hIgG1Fc(SSS)ΔK -T2m | **EPKSSDKTHTSPPSPAPE**LLGGPSVFLFPPKPKDTLMISRTPEVTCVVVDVSHEDPEVKFNWYVDGVEVHNAKTKPREEQYNSTYRVVSVLTVLHQDWLNGKEYKCKVSNKALPAPIEKTISKAKGQPREPQVYTLPPSRDELTKNQVSLTCLVKGFYPSDIAVEWESNGQPENNYKTTPPVLDSDGSFFLYSKLTVDKSRWQQGNVFSCSVMHEALHNHYTQKSLSLSPGIPPHVQKSVNNDMIVTDNNGAVKFPQLCKFCDVRFSTCDNQKSCMSNCSITSICEKPQEVCVAVWRKNDENITLETVCHDPKLPYHDFILEDAASPKCIMKEKKKPGETFFMCSCSSDECNDNIIFSEEYNTSNPD |
| hIgG1Fc(ΔC)ΔK -T2m | **APE**LLGGPSVFLFPPKPKDTLMISRTPEVTCVVVDVSHEDPEVKFNWYVDGVEVHNAKTKPREEQYNSTYRVVSVLTVLHQDWLNGKEYKCKVSNKALPAPIEKTISKAKGQPREPQVYTLPPSRDELTKNQVSLTCLVKGFYPSDIAVEWESNGQPENNYKTTPPVLDSDGSFFLYSKLTVDKSRWQQGNVFSCSVMHEALHNHYTQKSLSLSPGIPPHVQKSVNNDMIVTDNNGAVKFPQLCKFCDVRFSTCDNQKSCMSNCSITSICEKPQEVCVAVWRKNDENITLETVCHDPKLPYHDFILEDAASPKCIMKEKKKPGETFFMCSCSSDECNDNIIFSEEYNTSNPD |
| hIgG2Fc(SSCC)ΔK-T2m | **ERKSSVECPPCPAPP**VAGPSVFLFPPKPKDTLMISRTPEVTCVVVDVSHEDPEVQFNWYVDGVEVHNAKTKPREEQFNSTFRVVSVLTVVHQDWLNGKEYKCKVSNKGLPAPIEKTISKTKGQPREPQVYTLPPSREEMTKNQVSLTCLVKGFYPSDISVEWESNGQPENNYKTTPPMLDSDGSFFLYSKLTVDKSRWQQGNVFSCSVMHEALHNHYTQKSLSLSPGIPPHVQKSVNNDMIVTDNNGAVKFPQLCKFCDVRFSTCDNQKSCMSNCSITSICEKPQEVCVAVWRKNDENITLETVCHDPKLPYHDFILEDAASPKCIMKEKKKPGETFFMCSCSSDECNDNIIFSEEYNTSNPD |
| hIgG2Fc(SSSC)ΔK-T2m | **ERKSSVESPPCPAPP**VAGPSVFLFPPKPKDTLMISRTPEVTCVVVDVSHEDPEVQFNWYVDGVEVHNAKTKPREEQFNSTFRVVSVLTVVHQDWLNGKEYKCKVSNKGLPAPIEKTISKTKGQPREPQVYTLPPSREEMTKNQVSLTCLVKGFYPSDISVEWESNGQPENNYKTTPPMLDSDGSFFLYSKLTVDKSRWQQGNVFSCSVMHEALHNHYTQKSLSLSPGIPPHVQKSVNNDMIVTDNNGAVKFPQLCKFCDVRFSTCDNQKSCMSNCSITSICEKPQEVCVAVWRKNDENITLETVCHDPKLPYHDFILEDAASPKCIMKEKKKPGETFFMCSCSSDECNDNIIFSEEYNTSNPD |
| hIgG2Fc(SSSS)ΔK-T2m | **ERKSSVESPPSPAPP**VAGPSVFLFPPKPKDTLMISRTPEVTCVVVDVSHEDPEVQFNWYVDGVEVHNAKTKPREEQFNSTFRVVSVLTVVHQDWLNGKEYKCKVSNKGLPAPIEKTISKTKGQPREPQVYTLPPSREEMTKNQVSLTCLVKGFYPSDISVEWESNGQPENNYKTTPPMLDSDGSFFLYSKLTVDKSRWQQGNVFSCSVMHEALHNHYTQKSLSLSPGIPPHVQKSVNNDMIVTDNNGAVKFPQLCKFCDVRFSTCDNQKSCMSNCSITSICEKPQEVCVAVWRKNDENITLETVCHDPKLPYHDFILEDAASPKCIMKEKKKPGETFFMCSCSSDECNDNIIFSEEYNTSNPD |
| hIgG2Fc(ΔC)ΔK-T2m | **APP**VAGPSVFLFPPKPKDTLMISRTPEVTCVVVDVSHEDPEVQFNWYVDGVEVHNAKTKPREEQFNSTFRVVSVLTVVHQDWLNGKEYKCKVSNKGLPAPIEKTISKTKGQPREPQVYTLPPSREEMTKNQVSLTCLVKGFYPSDISVEWESNGQPENNYKTTPPMLDSDGSFFLYSKLTVDKSRWQQGNVFSCSVMHEALHNHYTQKSLSLSPGIPPHVQKSVNNDMIVTDNNGAVKFPQLCKFCDVRFSTCDNQKSCMSNCSITSICEKPQEVCVAVWRKNDENITLETVCHDPKLPYHDFILEDAASPKCIMKEKKKPGETFFMCSCSSDECNDNIIFSEEYNTSNPD |
| hIgG3Fc(SCC)ΔK-T2m | **EPKSSDTPPPCPRCPAPE**LLGGPSVFLFPPKPKDTLMISRTPEVTCVVVDVSHEDPEVQFKWYVDGVEVHNAKTKPREEQYNSTFRVVSVLTVLHQDWLNGKEYKCKVSNKALPAPIEKTISKTKGQPREPQVYTLPPSREEMTKNQVSLTCLVKGFYPSDIAVEWESSGQPENNYNTTPPMLDSDGSFFLYSKLTVDKSRWQQGNIFSCSVMHEALHNRFTQKSLSLSPGIPPHVQKSVNNDMIVTDNNGAVKFPQLCKFCDVRFSTCDNQKSCMSNCSITSICEKPQEVCVAVWRKNDENITLETVCHDPKLPYHDFILEDAASPKCIMKEKKKPGETFFMCSCSSDECNDNIIFSEEYNTSNPD |
| hIgG3Fc(SSC)ΔK-T2m | **EPKSSDTPPPSPRCPAPE**LLGGPSVFLFPPKPKDTLMISRTPEVTCVVVDVSHEDPEVQFKWYVDGVEVHNAKTKPREEQYNSTFRVVSVLTVLHQDWLNGKEYKCKVSNKALPAPIEKTISKTKGQPREPQVYTLPPSREEMTKNQVSLTCLVKGFYPSDIAVEWESSGQPENNYNTTPPMLDSDGSFFLYSKLTVDKSRWQQGNIFSCSVMHEALHNRFTQKSLSLSPGIPPHVQKSVNNDMIVTDNNGAVKFPQLCKFCDVRFSTCDNQKSCMSNCSITSICEKPQEVCVAVWRKNDENITLETVCHDPKLPYHDFILEDAASPKCIMKEKKKPGETFFMCSCSSDECNDNIIFSEEYNTSNPD |
| hIgG3Fc(SSS)ΔK-T2m | **EPKSSDTPPPSPRSPAPE**LLGGPSVFLFPPKPKDTLMISRTPEVTCVVVDVSHEDPEVQFKWYVDGVEVHNAKTKPREEQYNSTFRVVSVLTVLHQDWLNGKEYKCKVSNKALPAPIEKTISKTKGQPREPQVYTLPPSREEMTKNQVSLTCLVKGFYPSDIAVEWESSGQPENNYNTTPPMLDSDGSFFLYSKLTVDKSRWQQGNIFSCSVMHEALHNRFTQKSLSLSPGIPPHVQKSVNNDMIVTDNNGAVKFPQLCKFCDVRFSTCDNQKSCMSNCSITSICEKPQEVCVAVWRKNDENITLETVCHDPKLPYHDFILEDAASPKCIMKEKKKPGETFFMCSCSSDECNDNIIFSEEYNTSNPD |
| hIgG3Fc(ΔC)ΔK-T2m | **APE**LLGGPSVFLFPPKPKDTLMISRTPEVTCVVVDVSHEDPEVQFKWYVDGVEVHNAKTKPREEQYNSTFRVVSVLTVLHQDWLNGKEYKCKVSNKALPAPIEKTISKTKGQPREPQVYTLPPSREEMTKNQVSLTCLVKGFYPSDIAVEWESSGQPENNYNTTPPMLDSDGSFFLYSKLTVDKSRWQQGNIFSCSVMHEALHNRFTQKSLSLSPGIPPHVQKSVNNDMIVTDNNGAVKFPQLCKFCDVRFSTCDNQKSCMSNCSITSICEKPQEVCVAVWRKNDENITLETVCHDPKLPYHDFILEDAASPKCIMKEKKKPGETFFMCSCSSDECNDNIIFSEEYNTSNPD |
| hIgG4Fc(CC)ΔK-T2m | **ESKYGPPCPSCPAPE**FLGGPSVFLFPPKPKDTLMISRTPEVTCVVVDVSQEDPEVQFNWYVDGVEVHNAKTKPREEQFNSTYRVVSVLTVLHQDWLNGKEYKCKVSNKGLPSSIEKTISKAKGQPREPQVYTLPPSQEEMTKNQVSLTCLVKGFYPSDIAVEWESNGQPENNYKTTPPVLDSDGSFFLYSRLTVDKSRWQEGNVFSCSVMHEALHNHYTQKSLSLSLGIPPHVQKSVNNDMIVTDNNGAVKFPQLCKFCDVRFSTCDNQKSCMSNCSITSICEKPQEVCVAVWRKNDENITLETVCHDPKLPYHDFILEDAASPKCIMKEKKKPGETFFMCSCSSDECNDNIIFSEEYNTSNPD |
| hIgG4Fc(SC)ΔK-T2m | **ESKYGPPSPSCPAPE**FLGGPSVFLFPPKPKDTLMISRTPEVTCVVVDVSQEDPEVQFNWYVDGVEVHNAKTKPREEQFNSTYRVVSVLTVLHQDWLNGKEYKCKVSNKGLPSSIEKTISKAKGQPREPQVYTLPPSQEEMTKNQVSLTCLVKGFYPSDIAVEWESNGQPENNYKTTPPVLDSDGSFFLYSRLTVDKSRWQEGNVFSCSVMHEALHNHYTQKSLSLSLGIPPHVQKSVNNDMIVTDNNGAVKFPQLCKFCDVRFSTCDNQKSCMSNCSITSICEKPQEVCVAVWRKNDENITLETVCHDPKLPYHDFILEDAASPKCIMKEKKKPGETFFMCSCSSDECNDNIIFSEEYNTSNPD |
| hIgG4Fc(SS)ΔK-T2m | **ESKYGPPSPSSPAPE**FLGGPSVFLFPPKPKDTLMISRTPEVTCVVVDVSQEDPEVQFNWYVDGVEVHNAKTKPREEQFNSTYRVVSVLTVLHQDWLNGKEYKCKVSNKGLPSSIEKTISKAKGQPREPQVYTLPPSQEEMTKNQVSLTCLVKGFYPSDIAVEWESNGQPENNYKTTPPVLDSDGSFFLYSRLTVDKSRWQEGNVFSCSVMHEALHNHYTQKSLSLSLGIPPHVQKSVNNDMIVTDNNGAVKFPQLCKFCDVRFSTCDNQKSCMSNCSITSICEKPQEVCVAVWRKNDENITLETVCHDPKLPYHDFILEDAASPKCIMKEKKKPGETFFMCSCSSDECNDNIIFSEEYNTSNPD |
| hIgG4Fc(ΔC)ΔK-T2m | **APE**FLGGPSVFLFPPKPKDTLMISRTPEVTCVVVDVSQEDPEVQFNWYVDGVEVHNAKTKPREEQFNSTYRVVSVLTVLHQDWLNGKEYKCKVSNKGLPSSIEKTISKAKGQPREPQVYTLPPSQEEMTKNQVSLTCLVKGFYPSDIAVEWESNGQPENNYKTTPPVLDSDGSFFLYSRLTVDKSRWQEGNVFSCSVMHEALHNHYTQKSLSLSLGIPPHVQKSVNNDMIVTDNNGAVKFPQLCKFCDVRFSTCDNQKSCMSNCSITSICEKPQEVCVAVWRKNDENITLETVCHDPKLPYHDFILEDAASPKCIMKEKKKPGETFFMCSCSSDECNDNIIFSEEYNTSNPD |

**Supplemental Table S3:** Amino acid sequences of the ‘headless’ C-terminally hIgG1 and hIgG2 Fc-fused T22d35 constructs. hIgGFc heavy chain, blue; T22d35 sequence, black; natural linker sequences, black underlined; Fc hinge region, bold blue; paired cysteine residues, bold red; serine replaced cysteines, bold red underlined.

| hIgG1Fc(C)ΔK-T22d35 | **PPCPAPE**LLGGPSVFLFPPKPKDTLMISRTPEVTCVVVDVSHEDPEVKFNWYVDGVEVHNAKTKPREEQYNSTYRVVSVLTVLHQDWLNGKEYKCKVSNKALPAPIEKTISKAKGQPREPQVYTLPPSRDELTKNQVSLTCLVKGFYPSDIAVEWESNGQPENNYKTTPPVLDSDGSFFLYSKLTVDKSRWQQGNVFSCSVMHEALHNHYTQKSLSLSPGIPPHVQKSVNNDMIVTDNNGAVKFPQLCKFCDVRFSTCDNQKSCMSNCSITSICEKPQEVCVAVWRKNDENITLETVCHDPKLPYHDFILEDAASPKCIMKEKKKPGETFFMCSCSSDECNDNIIFSEEYNTSNPDIPPHVQKSVNNDMIVTDNNGAVKFPQLCKFCDVRFSTCDNQKSCMSNCSITSICEKPQEVCVAVWRKNDENITLETVCHDPKLPYHDFILEDAASPKCIMKEKKKPGETFFMCSCSSDECNDNIIFSEEYNTSNPD |
| --- | --- |
| hIgG1Fc(CC)ΔK-T22d35 | **DKTHTCPPCPAPE**LLGGPSVFLFPPKPKDTLMISRTPEVTCVVVDVSHEDPEVKFNWYVDGVEVHNAKTKPREEQYNSTYRVVSVLTVLHQDWLNGKEYKCKVSNKALPAPIEKTISKAKGQPREPQVYTLPPSRDELTKNQVSLTCLVKGFYPSDIAVEWESNGQPENNYKTTPPVLDSDGSFFLYSKLTVDKSRWQQGNVFSCSVMHEALHNHYTQKSZSZSPGIPPHVQKSVNNDMIVTDNNGAVKFPQLCKFCDVRFSTCDNQKSCMSNCSITSICEKPQEVCVAVWRKNDENITLETVCHDPKLPYHDFILEDAASPKCIMKEKKKPGETFFMCSCSSDECNDNIIFSEEYNTSNPDIPPHVQKSVNNDMIVTDNNGAVKFPQLCKFCDVRFSTCDNQKSCMSNCSITSICEKPQEVCVAVWRKNDENITLETVCHDPKLPYHDFILEDAASPKCIMKEKKKPGETFFMCSCSSDECNDNIIFSEEYNTSNPD |
| **Fc-T22d35**  hIgG2Fc(CC)ΔK-T22d35 | **VECPPCPAPP**VAGPSVFLFPPKPKDTLMISRTPEVTCVVVDVSHEDPEVQFNWYVDGVEVHNAKTKPREEQFNSTFRVVSVLTVVHQDWLNGKEYKCKVSNKGLPAPIEKTISKTKGQPREPQVYTLPPSREEMTKNQVSLTCLVKGFYPSDISVEWESNGQPENNYKTTPPMLDSDGSFFLYSKLTVDKSRWQQGNVFSCSVMHEALHNHYTQKSLSZSPGIPPHVQKSVNNDMIVTDNNGAVKFPQLCKFCDVRFSTCDNQKSCMSNCSITSICEKPQEVCVAVWRKNDENITLETVCHDPKLPYHDFILEDAASPKCIMKEKKKPGETFFMCSCSSDECNDNIIFSEEYNTSNPDIPPHVQKSVNNDMIVTDNNGAVKFPQLCKFCDVRFSTCDNQKSCMSNCSITSICEKPQEVCVAVWRKNDENITLETVCHDPKLPYHDFILEDAASPKCIMKEKKKPGETFFMCSCSSDECNDNIIFSEEYNTSNPD |

**Supplemental Table S4:** Amino acid sequences of the N-terminally hIgG1 and hIgG2 Fc-fused T2m and T22d35 constructs. hIgGFc heavy chain, blue; T22d35 sequence, black; natural linker sequences, black underlined; Fc hinge region, bold blue; paired cysteine residues, bold red; serine replaced cysteines, bold red underlined; glycine linker, bold italics.

| **T2m-Fc**  T2m-hIgG2Fc(CCCC)ΔK | IPPHVQKSVNNDMIVTDNNGAVKFPQLCKFCDVRFSTCDNQKSCMSNCSITSICEPQEVCVAVWRKNDENITLETVCHDPKLPYHDFILEDAASPKCIMKEKKKPGETFFMCSCSSDECNDNIIFSEEYNTSNPD**ERKCCVECPPCPAPP**VAGPSVFLFPPKPKDTLMISRTPEVTCVVVDVSHEDPEVQFNWYVDGVEVHNAKTKPREEQFNSTFRVVSVLTVVHQDWLNGKEYKCKVSNKGLPAPIEKTISKTKGQPREPQVYTLPPSREEMTKNQVSLTCLVKGFYPSDISVEWESNGQPENNYKTTPPMLDSDGSFFLYSKLTVDKSRWQQGNVFSCSVMHEALHNHYTQKSLSLSPG |
| --- | --- |
| T22d35-hIgG1Fc(CC)ΔK | IPPHVQKSVNNDMIVTDNNGAVKFPQLCKFCDVRFSTCDNQKSCMSNCSITSICEKPQEVCVAVWRKNDENITLETVCHDPKLPYHDFILEDAASPKCIMKEKKKPGETFFMCSCSSDECNDNIIFSEEYNTSNPDIPPHVQKSVNNDMIVTDNNGAVKFPQLCKFCDVRFSTCDNQKSCMSNCSITSICEKPQEVCVAVWRKNDENITLETVCHDPKLPYHDFILEDAASPKCIMKEKKKPGETFFMCSCSSDECNDNIIFSEEYNTSNPD**THTCPPCPAPE**LLGGPSVFLFPPKPKDTLMISRTPEVTCVVVDVSHEDPEVKFNWYVDGVEVHNAKTKPREEQYNSTYRVVSVLTVLHQDWLNGKEYKCKVSNKALPAPIEKTISKAKGQPREPQVYTLPPSRDELTKNQVSLTCLVKGFYPSDIAVEWESNGQPENNYKTTPPVLDSDGSFFLYSKLTVDKSRWQQGNVFSCSVMHEALHNHYTQKSLSLSPG |
| T22d35-hIgG1Fc(SCC)ΔK | IPPHVQKSVNNDMIVTDNNGAVKFPQLCKFCDVRFSTCDNQKSCMSNCSITSICEKPQEVCVAVWRKNDENITLETVCHDPKLPYHDFILEDAASPKCIMKEKKKPGETFFMCSCSSDECNDNIIFSEEYNTSNPDIPPHVQKSVNNDMIVTDNNGAVKFPQLCKFCDVRFSTCDNQKSCMSNCSITSICEKPQEVCVAVWRKNDENITLETVCHDPKLPYHDFILEDAASPKCIMKEKKKPGETFFMCSCSSDECNDNIIFSEEYNTSNPD**VEPKSSDKTHTCPPCPAPE**LLGGPSVFLFPPKPKDTLMISRTPEVTCVVVDVSHEDPEVKFNWYVDGVEVHNAKTKPREEQYNSTYRVVSVLTVLHQDWLNGKEYKCKVSNKALPAPIEKTISKAKGQPREPQVYTLPPSRDELTKNQVSLTCLVKGFYPSDIAVEWESNGQPENNYKTTPPVLDSDGSFFLYSKLTVDKSRWQQGNVFSCSVMHEALHNHYTQKSLSLSPG |
| T22d35-hIgG1Fc(GSL-CC)ΔK | IPPHVQKSVNNDMIVTDNNGAVKFPQLCKFCDVRFSTCDNQKSCMSNCSITSICEKPQEVCVAVWRKNDENITLETVCHDPKLPYHDFILEDAASPKCIMKEKKKPGETFFMCSCSSDECNDNIIFSEEYNTSNPDIPPHVQKSVNNDMIVTDNNGAVKFPQLCKFCDVRFSTCDNQKSCMSNCSITSICEKPQEVCVAVWRKNDENITLETVCHDPKLPYHDFILEDAASPKCIMKEKKKPGETFFMCSCSSDECNDNIIFSEEYNTSNPD***GGGSGGGSGGG*THTCPPCPAPE**LLGGPSVFLFPPKPKDTLMISRTPEVTCVVVDVSHEDPEVKFNWYVDGVEVHNAKTKPREEQYNSTYRVVSVLTVLHQDWLNGKEYKCKVSNKALPAPIEKTISKAKGQPREPQVYTLPPSRDELTKNQVSLTCLVKGFYPSDIAVEWESNGQPENNYKTTPPVLDSDGSFFLYSKLTVDKSRWQQGNVFSCSVMHEALHNHYTQKSLSLSPG |
| **T22d35-Fc**  T22d35-hIgG2Fc(CC)ΔK | IPPHVQKSVNNDMIVTDNNGAVKFPQLCKFCDVRFSTCDNQKSCMSNCSITSICEKPQEVCVAVWRKNDENITLETVCHDPKLPYHDFILEDAASPKCIMKEKKKPGETFFMCSCSSDECNDNIIFSEEYNTSNPDIPPHVQKSVNNDMIVTDNNGAVKFPQLCKFCDVRFSTCDNQKSCMSNCSITSICEKPQEVCVAVWRKNDENITLETVCHDPKLPYHDFILEDAASPKCIMKEKKKPGETFFMCSCSSDECNDNIIFSEEYNTSNPD**VECPPCPAPP**VAGPSVFLFPPKPKDTLMISRTPEVTCVVVDVSHEDPEVQFNWYVDGVEVHNAKTKPREEQFNSTFRVVSVLTVVHQDWLNGKEYKCKVSNKGLPAPIEKTISKTKGQPREPQVYTLPPSREEMTKNQVSLTCLVKGFYPSDISVEWESNGQPENNYKTTPPMLDSDGSFFLYSKLTVDKSRWQQGNVFSCSVMHEALHNHYTQKSLSLSPG |
| T22d35-hIgG2Fc(CCCC)ΔK | IPPHVQKSVNNDMIVTDNNGAVKFPQLCKFCDVRFSTCDNQKSCMSNCSITSICEKPQEVCVAVWRKNDENITLETVCHDPKLPYHDFILEDAASPKCIMKEKKKPGETFFMCSCSSDECNDNIIFSEEYNTSNPDIPPHVQKSVNNDMIVTDNNGAVKFPQLCKFCDVRFSTCDNQKSCMSNCSITSICEPQEVCVAVWRKNDENITLETVCHDPKLPYHDFILEDAASPKCIMKEKKKPGETFFMCSCSSDECNDNIIFSEEYNTSNPD**ERKCCVECPPCPAPP**VAGPSVFLFPPKPKDTLMISRTPEVTCVVVDVSHEDPEVQFNWYVDGVEVHNAKTKPREEQFNSTFRVVSVLTVVHQDWLNGKEYKCKVSNKGLPAPIEKTISKTKGQPREPQVYTLPPSREEMTKNQVSLTCLVKGFYPSDISVEWESNGQPENNYKTTPPMLDSDGSFFLYSKLTVDKSRWQQGNVFSCSVMHEALHNHYTQKSLSLSPG |

**Supplemental Table S5:** Amino acid sequences T2m and T22d35 sequences fused to the C- or N- terminus of a human IgG1 (blue) or murine IgG2a (blue italics) heavy chain. Cet light chain, blue underlined; T2m and T22d35 sequence, black; natural linker sequences, black underlined; Fc hinge region, bold blue; paired cysteine residues, bold red; serine replaced cysteines, bold red underlined; FC5V_H_H sequence, red.

| **Fc-T2m**  hIgG1Fc(CC)ΔK-T2m | **DKTHTCPPCPAPE**LLGGPSVFLFPPKPKDTLMISRTPEVTCVVVDVSHEDPEVKFNWYVDGVEVHNAKTKPREEQYNSTYRVVSVLTVLHQDWLNGKEYKCKVSNKALPAPIEKTISKAKGQPREPQVYTLPPSRDELTKNQVSLTCLVKGFYPSDIAVEWESNGQPENNYKTTPPVLDSDGSFFLYSKLTVDKSRWQQGNVFSCSVMHEALHNHYTQKSLSLSPGIPPHVQKSVNNDMIVTDNNGAVKFPQLCKFCDVRFSTCDNQKSCMSNCSITSICEKPQEVCVAVWRKNDENITLETVCHDPKLPYHDFILEDAASPKCIMKEKKKPGETFFMCSCSSDECNDNIIFSEEYNTSNPD |
| --- | --- |
| **Cet-T2m**  Heavy Chain | QVQLKQSGPGLVQPSQSLSITCTVSGFSLTNYGVHWVRQSPGKGLEWLGVIWSGGNTDYNTPFTSRLSINKDNSKSQVFFKMNSLQSNDTAIYYCARALTYYDYEFAYWGQGTLVTVSAASTKGPSVFPLAPSSKSTSGGTAALGCLVKDYFPEPVTVSWNSGALTSGVHTFPAVLQSSGLYSLSSVVTVPSSSLGTQTYICNVNHKPSNTKVDKRV**EPKSCDKTHTCPPCPAPE**LLGGPSVFLFPPKPKDTLMISRTPEVTCVVVDVSHEDPEVKFNWYVDGVEVHNAKTKPREEQYNSTYRVVSVLTVLHQDWLNGKEYKCKVSNKALPAPIEKTISKAKGQPREPQVYTLPPSRDELTKNQVSLTCLVKGFYPSDIAVEWESNGQPENNYKTTPPVLDSDGSFFLYSKLTVDKSRWQQGNVFSCSVMHEALHNHYTQKSLSLSPGKIPPHVQKSVNNDMIVTDNNGAVKFPQLCKFCDVRFSTCDNQKSCMSNCSITSICEKPQEVCVAVWRKNDENITLETVCHDPKLPYHDFILEDAASPKCIMKEKKKPGETFFMCSCSSDECNDNIIFSEEYNTSNPD |
| **Cet-T22d35**  Heavy Chain | QVQLKQSGPGLVQPSQSLSITCTVSGFSLTNYGVHWVRQSPGKGLEWLGVIWSGGNTDYNTPFTSRLSINKDNSKSQVFFKMNSLQSNDTAIYYCARALTYYDYEFAYWGQGTLVTVSAASTKGPSVFPLAPSSKSTSGGTAALGCLVKDYFPEPVTVSWNSGALTSGVHTFPAVLQSSGLYSLSSVVTVPSSSLGTQTYICNVNHKPSNTKVDKRV**EPKSCDKTHTCPPCPAPE**LLGGPSVFLFPPKPKDTLMISRTPEVTCVVVDVSHEDPEVKFNWYVDGVEVHNAKTKPREEQYNSTYRVVSVLTVLHQDWLNGKEYKCKVSNKALPAPIEKTISKAKGQPREPQVYTLPPSRDELTKNQVSLTCLVKGFYPSDIAVEWESNGQPENNYKTTPPVLDSDGSFFLYSKLTVDKSRWQQGNVFSCSVMHEALHNHYTQKSLSLSPGKIPPHVQKSVNNDMIVTDNNGAVKFPQLCKFCDVRFSTCDNQKSCMSNCSITSICEKPQEVCVAVWRKNDENITLETVCHDPKLPYHDFILEDAASPKCIMKEKKKPGETFFMCSCSSDECNDNIIFSEEYNTSNPDIPPHVQKSVNNDMIVTDNNGAVKFPQLCKFCDVRFSTCDNQKSCMSNCSITSICEKPQEVCVAVWRKNDENITLETVCHDPKLPYHDFILEDAASPKCIMKEKKKPGETFFMCSCSSDECNDNIIFSEEYNTSNPD |
| **Cet**  Light Chain | DILLTQSPVILSVSPGERVSFSCRASQSIGTNIHWYQQRTNGSPRLLIKYASESISGIPSRFSGSGSGTDFTLSINSVESEDIADYYCQQNNNWPTTFGAGTKLELKRTVAAPSVFIFPPSDEQLKSGTASVVCLLNNFYPREAKVQWKVDNALQSGNSQESVTEQDSKDSTYSLSSTLTLSKADYEKHKVYACEVTHQGLSSPVTKSFNRGEC |
| **FC5-Fc-T2m**  FC5-mIgG2aFc-T2m | DVQLQASGGGLVQAGGSLRLSCAASGFKITHYTMGWFRQAPGKEREFVSRITWGGDNTFYSNSVKGRFTISRDNAKNTVYLQMNSLKPEDTADYYCAAGSTSTATPLRVDYWGKGTQVTVSS***EPRGPTIKPCPPCKCPAPN****LLGGPSVFIFPPKIKDVLMISLSPIVTCVVVDVSEDDPDVQISWFVNNVEVHTAQTQTHREDYNSTLRVVSALPIQHQDWMSGKEFKCKVNNKDLPAPIERTISKPKGSVRAPQVYVLPPPEEEMTKKQVTLTCMVTDFMPEDIYVEWTNNGKTELNYKNTEPVLDSDGSYFMYSKLRVEKKNWVERNSYSCSVVHEGLHNHHTTKSFSRTPGTG*IPPHVQKSVNNDMIVTDNNGAVKFPQLCKFCDVRFSTCDNQKSCMSNCSITSICEKPQEVCVAVWRKNDENITLETVCHDPKLPYHDFILEDAASPKCIMKEKKKPGETFFMCSCSSDECNDNIIFSEEYNTSNPD |
| **FC5-Fc-T22d35**  FC5-mIgG2aFc-T22d35 | DVQLQASGGGLVQAGGSLRLSCAASGFKITHYTMGWFRQAPGKEREFVSRITWGGDNTFYSNSVKGRFTISRDNAKNTVYLQMNSLKPEDTADYYCAAGSTSTATPLRVDYWGKGTQVTVSS***EPRGPTIKPCPPCKCPAPN****LLGGPSVFIFPPKIKDVLMISLSPIVTCVVVDVSEDDPDVQISWFVNNVEVHTAQTQTHREDYNSTLRVVSALPIQHQDWMSGKEFKCKVNNKDLPAPIERTISKPKGSVRAPQVYVLPPPEEEMTKKQVTLTCMVTDFMPEDIYVEWTNNGKTELNYKNTEPVLDSDGSYFMYSKLRVEKKNWVERNSYSCSVVHEGLHNHHTTKSFSRTPGTG*IPPHVQKSVNNDMIVTDNNGAVKFPQLCKFCDVRFSTCDNQKSCMSNCSITSICEKPQEVCVAVWRKNDENITLETVCHDPKLPYHDFILEDAASPKCIMKEKKKPGETFFMCSCSSDECNDNIIFSEEYNTSNPDIPPHVQKSVNNDMIVTDNNGAVKFPQLCKFCDVRFSTCDNQKSCMSNCSITSICEKPQEVCVAVWRKNDENITLETVCHDPKLPYHDFILEDAASPKCIMKEKKKPGETFFMCSCSSDECNDNIIFSEEYNTSNPD |

**Supplemental Table S6:** Amino acid sequences of the designed D10-Fc-T2m constructs. hIgGFc heavy chain, blue; T2m sequence, black; natural linker sequences, black underlined; Fc hinge region, bold blue; paired cysteine residues, bold red; glycine linker, bold italics; D10 sequence, red.

| **D10-Fc-T2m**  D10-hIgG1Fc**(**CC)ΔK-T2m | DDDDDDDDDD**DKTHTCPPCPAPE**LLGGPSVFLFPPKPKDTLMISRTPEVTCVVVDVSHEDPEVKFNWYVDGVEVHNAKTKPREEQYNSTYRVVSVLTVLHQDWLNGKEYKCKVSNKALPAPIEKTISKAKGQPREPQVYTLPPSRDELTKNQVSLTCLVKGFYPSDIAVEWESNGQPENNYKTTPPVLDSDGSFFLYSKLTVDKSRWQQGNVFSCSVMHEALHNHYTQKSLSLSPGIPPHVQKSVNNDMIVTDNNGAVKFPQLCKFCDVRFSTCDNQKSCMSNCSITSICEKPQEVCVAVWRKNDENITLETVCHDPKLPYHDFILEDAASPKCIMKEKKKPGETFFMCSCSSDECNDNIIFSEEYNTSNPD |
| --- | --- |
| D10-GSL-hIgG1FcΔK**(**CC)-T2m | DDDDDDDDDD***GGGS*DKTHTCPPCPAPE**LLGGPSVFLFPPKPKDTLMISRTPEVTCVVVDVSHEDPEVKFNWYVDGVEVHNAKTKPREEQYNSTYRVVSVLTVLHQDWLNGKEYKCKVSNKALPAPIEKTISKAKGQPREPQVYTLPPSRDELTKNQVSLTCLVKGFYPSDIAVEWESNGQPENNYKTTPPVLDSDGSFFLYSKLTVDKSRWQQGNVFSCSVMHEALHNHYTQKSLSLSPGIPPHVQKSVNNDMIVTDNNGAVKFPQLCKFCDVRFSTCDNQKSCMSNCSITSICEKPQEVCVAVWRKNDENITLETVCHDPKLPYHDFILEDAASPKCIMKEKKKPGETFFMCSCSSDECNDNIIFSEEYNTSNPD |
| D10-hIgG1FcΔK**(**C)-T2m | DDDDDDDDDD**PPCPAPE**LLGGPSVFLFPPKPKDTLMISRTPEVTCVVVDVSHEDPEVKFNWYVDGVEVHNAKTKPREEQYNSTYRVVSVLTVLHQDWLNGKEYKCKVSNKALPAPIEKTISKAKGQPREPQVYTLPPSRDELTKNQVSLTCLVKGFYPSDIAVEWESNGQPENNYKTTPPVLDSDGSFFLYSKLTVDKSRWQQGNVFSCSVMHEALHNHYTQKSLSLSPGIPPHVQKSVNNDMIVTDNNGAVKFPQLCKFCDVRFSTCDNQKSCMSNCSITSICEKPQEVCVAVWRKNDENITLETVCHDPKLPYHDFILEDAASPKCIMKEKKKPGETFFMCSCSSDECNDNIIFSEEYNTSNPD |
| D10-GSL-hIgG1FcΔK**(**C)-T2m | DDDDDDDDDD***GGGSGGGS*PPCPAPE**LLGGPSVFLFPPKPKDTLMISRTPEVTCVVVDVSHEDPEVKFNWYVDGVEVHNAKTKPREEQYNSTYRVVSVLTVLHQDWLNGKEYKCKVSNKALPAPIEKTISKAKGQPREPQVYTLPPSRDELTKNQVSLTCLVKGFYPSDIAVEWESNGQPENNYKTTPPVLDSDGSFFLYSKLTVDKSRWQQGNVFSCSVMHEALHNHYTQKSLSLSPGIPPHVQKSVNNDMIVTDNNGAVKFPQLCKFCDVRFSTCDNQKSCMSNCSITSICEKPQEVCVAVWRKNDENITLETVCHDPKLPYHDFILEDAASPKCIMKEKKKPGETFFMCSCSSDECNDNIIFSEEYNTSNPD |

**Supplemental Table S7:** Overview of the small-scale (3x3 mL) transient expressions (before and after Protein A purification) of the N-terminal Fc-fused T2m variants in CHO-3E7 cells.

|  | |  | **After Expression** | | | **After Purification** | | | |
| --- | --- | --- | --- | --- | --- | --- | --- | --- | --- |
| **ID** |  | **MW** | **Volume (mL)** | **Amount (mg)** | **Conc (mg/mL)** | **Vol (mL)** | **Amount (mg)** | **Conc. (mg/mL)** | **Buffer** |
| hIgG1Fc(SCC)ΔK-T2m | | 82685.4 | 3x 3 (pooled) | 0.372 | 0.041 | 0.3 | 0.306 | 1.02 | 0.09M NaCitrate Buffer + ~0.14M Tris pH6.5-7.5 |
| hIgG1Fc(SSC)ΔK-T2m | | 82635.4 | 3x 3 (pooled) | 0.395 | 0.044 | 0.3 | 0.312 | 1.04 |  |
| hIgG1Fc(SSS)ΔK-T2m | | 82603.3 | 3x 3 (pooled) | 0.328 | 0.036 | 0.3 | 0.258 | 0.86 |  |
| hIgG1Fc(ΔC)ΔK-T2m | | 79449.9 | 3x 3 (pooled) | 0.329 | 0.037 | 0.3 | 0.264 | 0.88 |  |
| hIgG2Fc(SSCC)ΔK-T2m | | 81886.7 | 3x 3 (pooled) | 0.162 | 0.018 | 0.3 | 0.147 | 0.49 |  |
| hIgG2Fc(SSSC)ΔK-T2m | | 81854.6 | 3x 3 (pooled) | 0.176 | 0.020 | 0.3 | 0.156 | 0.52 |  |
| hIgG2Fc(SSSS)ΔK-T2m | | 81822.5 | 3x 3 (pooled) | 0.235 | 0.026 | 0.3 | 0.198 | 0.66 |  |
| hIgG2Fc(ΔC)ΔK-T2m | | 79259.7 | 3x 3 (pooled) | 0.255 | 0.028 | 0.3 | 0.213 | 0.71 |  |
| hIgG3Fc(SCC)ΔK-T2m | | 82685.4 | 3x 3 (pooled) | 0.148 | 0.014 | 0.3 | 0.078 | 0.26 |  |
| hIgG3Fc(SSC)ΔK-T2m | | 82635.4 | 3x 3 (pooled) | 0.157 | 0.011 | 0.3 | 0.069 | 0.23 |  |
| hIgG3Fc(SSS)ΔK-T2m | | 82603.3 | 3x 3 (pooled) | 0.133 | 0.012 | 0.3 | 0.069 | 0.23 |  |
| hIgG3Fc(ΔC)ΔK-T2m | | 79449.9 | 3x 3 (pooled) | 0.145 | 0.015 | 0.3 | 0.09 | 0.3 |  |
| hIgG4Fc(CC)ΔK-T2m | | 81886.7 | 3x 3 (pooled) | Too low for purification | | | | |  |
| hIgG4Fc(SC)ΔK-T2m | | 81854.6 | 3x 3 (pooled) | Too low for purification | | | | |  |
| hIgG4Fc(SS)ΔK-T2m | | 81822.5 | 3x 3 (pooled) | Too low for purification | | | | |  |
| hIgG4Fc(ΔC)ΔK-T2m | | 79259.7 | 3x 3 (pooled) | Too low for purification | | | | |  |

**Supplemental Table S8:** Overview of the titers and purity of the various N-and C-terminal hIgFc monovalent T2m and T22d35 fusions purified from stable CHO55E1 cell pools (0.5L, 10 days post-transfection).

|  | **T2m-hIgG2Fc (CCCC)ΔK** | **T22d35-hIgG2Fc (CC)ΔK** | **T22d35-hIgG1Fc (CC)ΔK** | **T22d35-hIgG1Fc (SCC)ΔK** | **T22d35-hIgG1Fc (GSL-CC)ΔK** | **hIgG1Fc (C)ΔK-T22d35** | **hIgG1Fc (CC)ΔK-T22d35** | **hIgG2F(CC)ΔK-T22d35** |
| --- | --- | --- | --- | --- | --- | --- | --- | --- |
| **AVG cell viability at harvest (%)** | 69.4 | 97 | 97.6 | 95.1 | 98 | 94.3 | 95.7 | 90.3 |
| **Final volume after 0.2 μm filtration (L)** | 0.65 | 0.65 | 0.65 | 0.65 | 0.65 | 0.65 | 0.65 | 0.65 |
| **Titer by pA-HPLC (mg/L)** | 212 | 214 | 556 | 353 | 526 | 260 | 339 | 119 |
| **Max expected yield (mg)** | 150 | 150 | 150 | 150 | 150 | 150 | 150 | 150 |
| **Final yield (mg)** | 67.34 | 62.17 | 81.64 | 81.74 | 74.84 | 70.95 | 81.49 | 48.73 |
| **Recovery (%)** | 44.89 | 41.45 | 54.43 | 54.49 | 49.89 | 47.3 | 54.33 | 31.49 |
| **Titer after protA purification (mg/mL)** | 212 | 214 | 556 | 353 | 526 | 260 | 339 | 119 |
| **Purity by UPLC-SEC (%)** | 99.4 | 99.65 | 99.3 | 98.8 | 99.45 | 86.95 | 90.45 | 96.75 |

**Supplemental Table S9:** Overview of the titers and purity of the various C-terminal T2m and T22d35 bifunctional fusions. Fusions were expressed by transient expression in CHO3E7 cells (harvest at 10 days post-transfection), except for the Cet-T2m and Cet-T22d35 (indicated with ‘*’), which were purified from stably transfected CHO55E1 cell pools (harvest at 15 days post-transfection),

|  | **Expression volume (L)** | **Yield (mg)** | **Yield (mg/L)** | **Monomer (%)** |
| --- | --- | --- | --- | --- |
| **Cet-T2m*** | 11 | 533 | 48 | 99.79 |
| **Cet-T22d35*** | 10 | 499 | 49 | 99.42 |
| **Her-T22d35** | 1 | 86 | 86 | 98.41 |
| **Ava-T22d35** | 1 | 41 | 41 | 99.23 |
| **Syn-T22d35** | 1 | 47 | 47 | 98.11 |
| **FC5-mFc-T2m** | 1 | 108 | 108 | 94.89 |
| **FC5-mFc-T22d35** | 1 | 37 | 37 | 98.12 |
| **D10-hFc-T2m** | 1 | 102 | 102 | 99.78 |
